# Do Small Molecules Activate the TrkB Receptor in the Same Manner as BDNF? Limitations of Published TrkB Low Molecular Agonists and Screening for Novel TrkB Orthosteric Agonists

**DOI:** 10.3390/ph14080704

**Published:** 2021-07-21

**Authors:** Piotr Pankiewicz, Marcin Szybiński, Katarzyna Kisielewska, Filip Gołębiowski, Patryk Krzemiński, Izabela Rutkowska-Włodarczyk, Rafał Moszczyński-Pętkowski, Lidia Gurba-Bryśkiewicz, Monika Delis, Krzysztof Mulewski, Damian Smuga, Jakub Dominowski, Artur Janusz, Michał Górka, Krzysztof Abramski, Agnieszka Napiórkowska, Marcin Nowotny, Krzysztof Dubiel, Katarzyna Kalita, Maciej Wieczorek, Jerzy Pieczykolan, Mikołaj Matłoka

**Affiliations:** 1 Celon Pharma Innovative Drugs Research & Development Department, Celon Pharma S.A., 05-152 Kazun Nowy, Poland; piotr.pankiewicz@celonpharma.com (P.P.); marcin.szybinski@celonpharma.com (M.S.); katarzyna.switon@gmail.com (K.K.); filip.golebiowski@gmail.com (F.G.); patrick-k@wp.pl (P.K.); izabela.rutkowska@masdiag.pl (I.R.-W.); rafal.moszczynski@celonpharma.com (R.M.-P.); lidia.gurba@celonpharma.com (L.G.-B.); monika.delis@celonpharma.com (M.D.); kmulewski91@gmail.com (K.M.); damian.smuga@celonpharma.com (D.S.); jakub.dominowski@celonpharma.com (J.D.); artur.janusz@celonpharma.com (A.J.); michal.gorka@celonpharma.com (M.G.); krzysztof.abramski@celonpharma.com (K.A.); krzysztof.dubiel@celonpharma.com (K.D.); maciej.wieczorek@celonpharma.com (M.W.); jerzy.pieczykolan@celonpharma.com (J.P.); 2 Postgraduate School of Molecular Medicine, Medical University of Warsaw, 02-091 Warsaw, Poland; 3 Structural Biology Center, International Institute of Molecular and Cell Biology, 02-109 Warsaw, Poland; anapiorkowska@leaderna.com (A.N.); mnowotny@iimcb.gov.pl (M.N.); 4 Laboratory of Protein Structure, International Institute of Molecular and Cell Biology, 02-109 Warsaw, Poland; 5 Laboratory of Neurobiology, Nencki-EMBL Partnership for Neural Plasticity and Brain Disorders—BRAINCITY, Nencki Institute of Experimental Biology PAS, 02-093 Warsaw, Poland; k.kalita@nencki.edu.pl

**Keywords:** TrkB agonist, drug discovery, 7,8-DHF

## Abstract

TrkB is a tyrosine kinase receptor that is activated upon binding to brain-derived neurotrophic factor (BDNF). To date, the search for low-molecular-weight molecules mimicking BDNF’s action has been unsuccessful. Several molecules exerting antidepressive effects in vivo, such as 7,8-DHF, have been suggested to be TrkB agonists. However, more recent publications question this hypothesis. In this study, we developed a set of experimental procedures including the evaluation of direct interactions, dimerization, downstream signaling, and cytoprotection in parallel with physicochemical and ADME methods to verify the pharmacology of 7,8-DHF and other potential reference compounds, and perform screening for novel TrkB agonists. 7,8 DHF bound to TrkB with K_d_ = 1.3 μM; however, we were not able to observe any other activity against the TrkB receptor in SN56 T48 and differentiated SH-SY5Y cell lines. Moreover, the pharmacokinetic and pharmacodynamic effects of 7,8-DHF at doses of 1 and 50 mg/kg were examined in mice after i.v and oral administration, respectively. The poor pharmacokinetic properties and lack of observed activation of TrkB-dependent signaling in the brain confirmed that 7,8-DHF is not a relevant tool for studying TrkB activation in vivo. The binding profile for 133 molecular targets revealed a significant lack of selectivity of 7,8-DHF, suggesting a distinct functional profile independent of interaction with TrkB. Additionally, a compound library was screened in search of novel low-molecular-weight orthosteric TrkB agonists; however, we were not able to identify reliable drug candidates. Our results suggest that published reference compounds including 7,8-DHF do not activate TrkB, consistent with canonical dogma, which indicates that the reported pharmacological activity of these compounds should be interpreted carefully in a broad functional context.

## 1. Introduction

Brain-derived neurotrophic factor (BDNF), an endogenous tropomyosin receptor kinase B (TrkB) receptor agonist, is a member of the neurotrophin family and controls many biological processes in neurons. At the cellular level, BDNF acts as a key regulator of neurite outgrowth, synaptic plasticity, and long-term potentiation in the central nervous system [1,2]. BDNF has antiapoptotic and neurotrophic properties affecting the viability and physiological condition of neurons [3,4]. Based on canonical knowledge, BDNF triggers TrkB dimerization, which leads to the autophosphorylation of tyrosine residues on the intracellular domain of the receptor. Consequently, three intracellular signaling pathways, relating to PLCγ1, MAPK, and PI3K kinases, may become activated [5]. Nevertheless, TrkB is also able to signal as a monomer, activating only the ERK_1/2_-dependent pathway [6]. The disruption of BDNF and TrkB homeostasis is involved in the pathophysiology of depression. Clinical evidence shows that patients suffering from depression demonstrate decreased BDNF levels in the hippocampus [7]. Moreover, reduced expression of the TrkB receptor in depressive patients with suicide risk and suicidal ideation has also been observed [8]. Furthermore, BDNF appears to be a key transducer in mediating the therapeutic effects of known antidepressants, such as selective serotonin reuptake inhibitors (SSRIs) and ketamine, by inducing changes in neuroplasticity [9]. The currently available antidepressants have many limitations. These may include side effects such as insomnia, sleepiness, and weight gain [10], and limited efficacy in drug-resistant forms of the disease [11]. As a result, targeting the BDNF–TrkB pathway with small molecular compounds may lead to a new class of therapeutics for the treatment of depression.

To date, several low-molecular-weight compounds have been postulated to act as TrkB agonists. The best characterized and most investigated is 7,8-dihydroxyflavone (7,8-DHF), first described by Jang and colleagues in 2010 [12]. 7,8-DHF exerted antidepressive activity in vivo in several animal models of depression [13,14,15,16]. Further studies on 7,8-DHF and other molecules have demonstrated BDNF-like activity, including neuroprotective, neurotrophic, and neuroplasticity-inducing properties [13,17,18,19,20,21,22,23,24]. However, results published by Todd et al., in 2014 and Boltaev et al., in 2017 contradict the postulated mechanism of action—according to their data, molecules described as low-molecular-weight TrkB agonists demonstrated limited activity through the TrkB receptor [25,26]. Here, we engaged several molecular assays to investigate the effects of reported low-molecular-weight agonists of TrkB on direct binding, dimerization potential, downstream signaling, and cytoprotective properties. 7,8-DHF was additionally evaluated in vivo, and the selectivity of the compound was determined. Furthermore, the screening platform, in conjunction with physicochemical and preliminary ADME properties, was applied to search for novel TrkB agonists.

## 2. Results

### 2.1. Primary Screening

#### 2.1.1. Identification of Molecules Binding to TrkB Using MicroScale Thermophoresis

**Figure 1:**
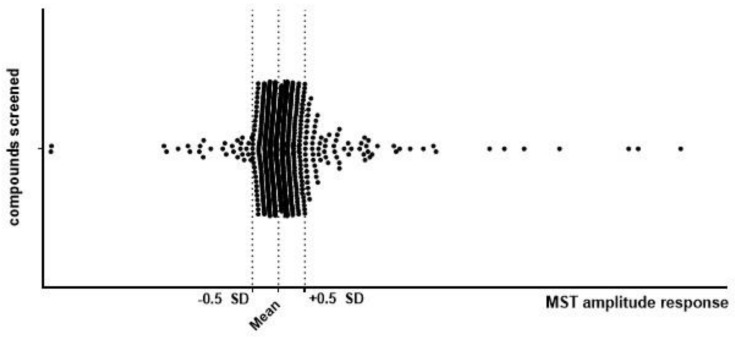
Results of primary screening using the MST technique. Screening of 987 compounds was performed by examination of signal amplitude between the unbound and bound state of the molecule and the exTrkB receptor. Compounds were screened in single-point MST at concentrations of 0.5 mM for small molecular compounds and 20 nM exTrkB. Compounds with amplitudes greater than 0.5 SD were selected for further analysis.

The MicroScale Thermophoresis (MST) technique was chosen to identify molecules able to interact with the extracellular domain of the TrkB receptor (exTrkB). In the first screening phase, 987 compounds were tested for binding to the labeled exTrkB receptor and the MST signal was recorded (Figure 1). The acquired results were in the range of −9.6 to 46.6. One hundred and eighty-one compounds with amplitudes greater than 0.5 standard deviations (SDs) were selected for further analysis.

#### 2.1.2. Determination of Dissociation Constant (K_d_) by MST

**Figure 2:**
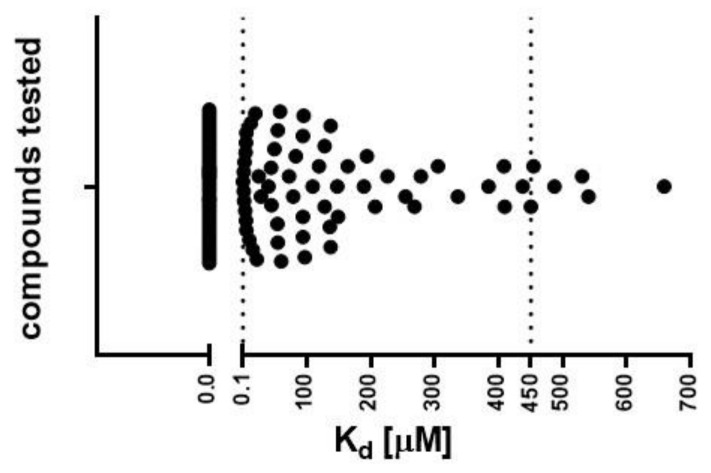
Binding affinity values (K_d_) determined using the MST technique. Threshold (dotted lines) for compound selection was set from 0.1 to 450 μM. Fifty-nine molecules from the pool of 113 tested compounds presented K_d_ values in the range 0.1–450 μM. Non-binding compounds are indicated on the graph as K_d_ = 0 μM.

In the next step, we determined the K_d_ values of selected compounds (including references) to select molecules with the best binding affinity. Only molecules soluble in DMSO at a concentration of 10 mM were examined, leading to the evaluation of 113 molecules. The K_d_ values were successfully determined for 65 compounds (Figure 2), ranging from 0.1 to 658 μM (Table S1). Binding to the exTrkB receptor was not observed for several molecules that were described in the literature as TrkB agonists, including 7,8,3′-trihydroxyflavone (7,8,3′-THF), 4′-dimethylamino-7,8-DHF, GSB-106, LM22-A4, LM22-B10, HIOC, *N*-acetylserotonin (NAS), 8-hydroxy-3-(4′-methoxyphenyl)-isocumarin (Isocumarin), and NSI-189. Fifty-nine molecules (K_d_ = 0.1–450 μM) were qualified for further studies.

### 2.2. Secondary Screening

#### TrkB Receptor Activation in a Functional Model

**Figure 3:**
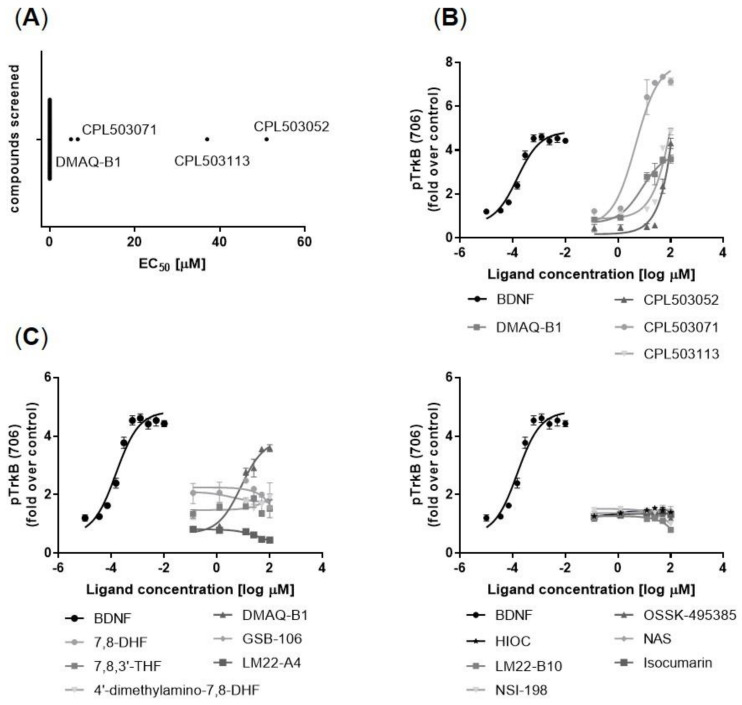
Orthosteric activity of compounds measured as the fold change of TrkB phosphorylation (Y706), determined by enzyme-linked fixed-cell immunoassay (ELFI) method in the SN56 T48 cell line. (**A**) Screening of 59 molecules led to the identification of 4 compounds with orthosteric activity against TrkB. Molecules with no orthosteric activity are marked on the graph as EC_50_ = 0 μM; (**B**) Representative dose–response curves for active TrkB agonist compounds; (**C**) Lack of activity against the TrkB receptor for several compounds described in the literature as TrkB agonists. Data are presented as mean ± SEM, *n* = 3.

Subsequently, 59 compounds selected in the primary screening were tested functionally to evaluate their ability to induce TrkB receptor phosphorylation (Tyr706) in vitro (Figure 3A). BDNF induces TrkB phosphorylation with a half maximal effective concentration (EC_50_) = 3.7 nM (Figure 3B). The following compounds were identified as TrkB orthosteric activators (Figure 3B): DMAQ-B1 (EC_50_ = 5.6 μM), CPL503052 (EC_50_ = 2.6 μM), CPL503071 (EC_50_ = 6.6 μM), and CPL503113 (EC_50_ = 44.2 μM). However, remaining compounds postulated to act as TrkB agonists, including 7,8-DHF, failed to show any activity in this assay and did not induce TrkB phosphorylation in vitro (Figure 3C). Due to the lack of orthosteric activity observed for reference compounds, the ability to induce TrkB phosphorylation was analyzed in a positive allosteric mode (PAM) experiment. All of the tested molecules were not able to positively modulate BDNF’s activity on TrkB (Figure S1).

**Figure 4:**
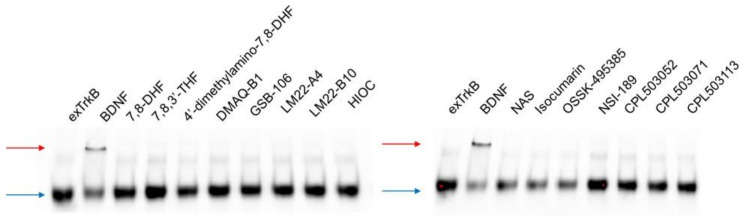
Evaluation of ligand-induced TrkB dimerization by native electrophoresis. The exTrkB receptor was incubated with tested compounds (stoichiometry ratio, 1:1). Dimerization of exTrkB was monitored by sample separation in non-reducing conditions. The upper band (red arrow) corresponds to exTrkB dimers induced by ligand binding; the lower band (blue arrow) corresponds to exTrkB monomers.

Moreover, native electrophoresis was performed to evaluate the dimerization-inducing properties of selected compounds. As shown in Figure 4, BDNF was able to induce exTrkB dimerization. No effect on ligand-provoked exTrkB dimerization was observed for low-molecular-weight compounds.

A summary of the binding to TrkB, and its activation (EC_50_ and PAM activity) and dimerization obtained for reference compounds and active molecules identified in the screening is presented in Table 1.

### 2.3. Evaluation of Compounds’ Activity on TrkB Downstream Signaling

#### 2.3.1. SN56 T48 Cell Line

**Figure 5:**
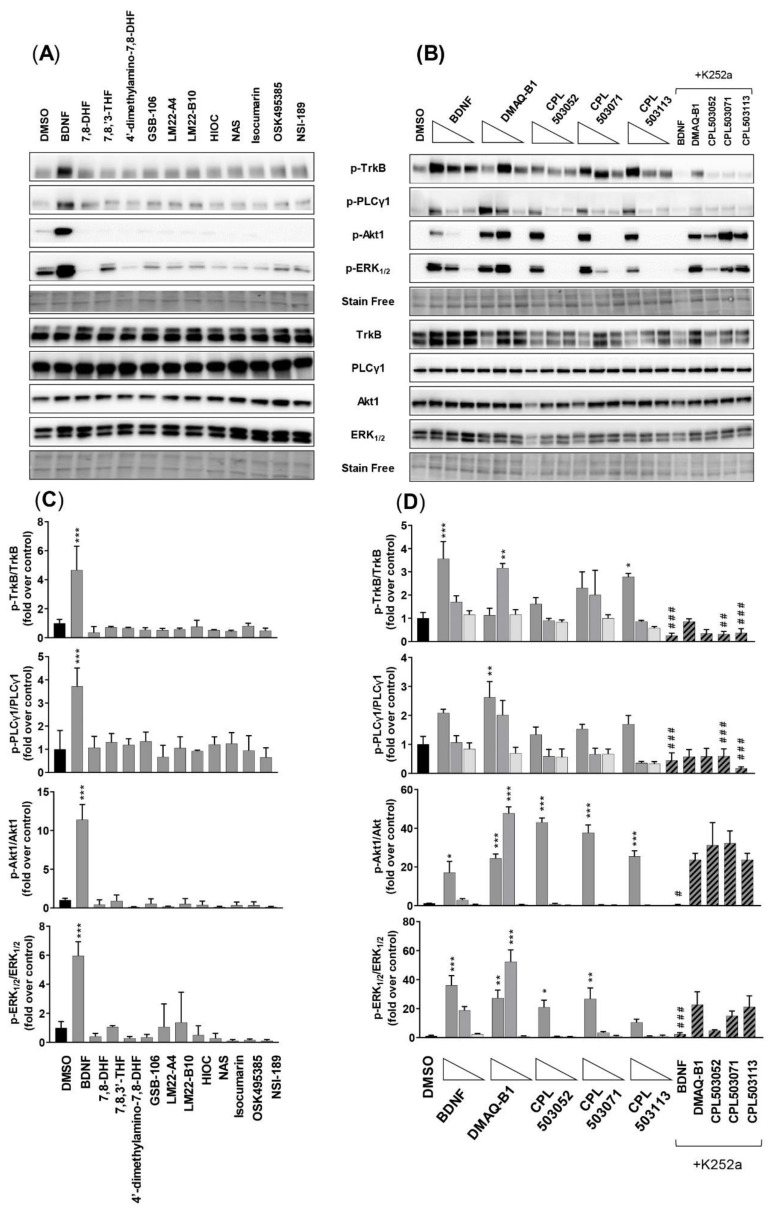
Evaluation of compounds’ activity on TrkB and downstream protein phosphorylation in the SN56 T48 cell line. (**A**) Representative immunoblots for proteins of interest (p-TrkB, p-PLCγ1, p-Akt1, and p-ERK1/2). Cells were treated with BDNF (100 ng/mL) or reference compounds (100 μM) for 15 min; (**B**) Representative immunoblots for proteins of interest. Cells were treated with BDNF (100 ng/mL; 10 ng/mL; 1 ng/mL) or tested compounds (100 μM; 10 μM; 1 μM) for 15 min. K252a (1 μM) was used 15 min before treatment with tested compounds (100 μM); (**C**,**D**) Densitometry analysis of immunoblots. Bars represent mean ± SEM, *n* = 3. Data were analyzed with one-way ANOVA followed by Dunnett’s post hoc test. * *p* < 0.05, ** *p* < 0.01 and *** *p* < 0.001 compared to the DMSO-treated group. # *p* < 0.05, ## *p* < 0.01 and ### *p* < 0.001 compared to the compound-treated group.

The effect on the phosphorylation of downstream proteins (the phosphorylation of PLCγ1 on Tyr783, phosphorylation of Akt1 on Ser473, and phosphorylation of ERK_1/2_ on Thr202/Tyr204) was evaluated in SN56 T48 cells using an immunoblotting technique. Cells treated with BDNF demonstrated significantly increased phosphorylation of PLCγ1, Akt1, and ERK_1/2_, with simultaneous induction of TrkB phosphorylation in a dose-dependent manner (Figure 5A–D). Western blot analysis confirmed the lack of TrkB phosphorylation for 7,8-DHF, which was previously assessed by the ELFI method (Figure 3), where concentrations of 100 μM did not induce the phosphorylation of TrkB and downstream proteins (Figure 5A,C). The lack of effect was observed for all the reference compounds except DMAQ-B1 (Figure 5A,C). Cells treated with CPL503052, CPL503071, and CPL503113, active compounds identified in the screening phase, demonstrated the activation of the TrkB receptor and phosphorylation of relevant downstream proteins (Figure 5B,D). However, the inhibition of TrkB kinase by K252a in the case of these compounds did not repress the activation of downstream proteins (Akt1 and ERK_1/2_), which was observed for BDNF (Figure 5B,D).

#### 2.3.2. SH-SY5Y Cell Line

**Figure 6:**
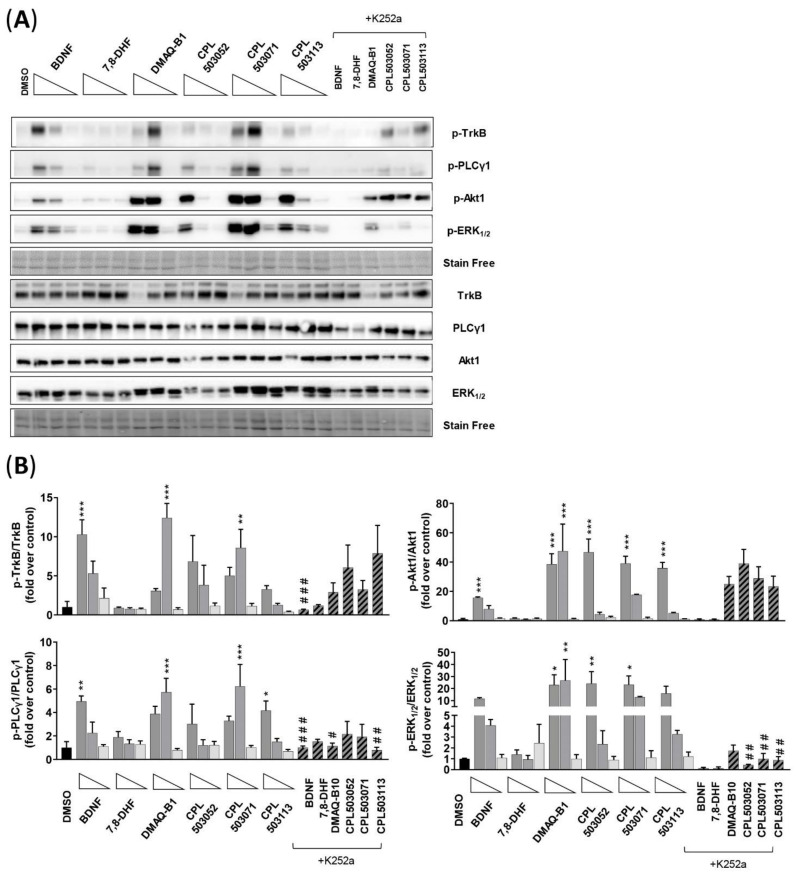
Evaluation of the impact of selected compounds on TrkB and downstream protein phosphorylation in the RA-differentiated SH-SY5Y cell line. (**A**) Representative immunoblots for proteins of interest (p-TrkB, p-PLCγ1, p-Akt1, and p-ERK_1/2_). Cells were treated with BDNF (100 ng/mL; 10 ng/mL; 1 ng/mL) and tested compounds (100 μM; 10 μM; 1 μM) for 15 min. K252a (1 μM) was used 15 min before treatment with tested compounds (100 μM); (**B**) Densitometry analysis of immunoblots. Bars represent mean ± SEM, *n* = 3. Data were analyzed with one-way ANOVA followed by Dunnett’s post hoc test. * *p* < 0.05, ** *p* < 0.01, and *** *p* < 0.001 compared to DMSO-treated group. # *p* < 0.05, ## *p* < 0.01, and ### *p* < 0.001 compared to compound-treated group.

Due to the lack of activity for 7,8-DHF and other reported low-molecular-weight TrkB receptor agonists in SN56 T48 cells, we subsequently evaluated the activity of these compounds using a differentiated SH-SY5Y model. The SH-SY5Y cell line differentiated by retinoic acid (RA) is a widely used neuron-like model in neurobiological research [27]. Moreover, retinoic acid treatment induces TrkB overexpression, as confirmed by our Western blot analysis (Figure S2). As expected, treatment with BDNF induced the TrkB receptor and phosphorylation of downstream proteins (PLCγ1 Tyr783, Akt1 Ser473, and ERK_1/2_ Thr202/Tyr204) (Figure 6A,B). The treatment of cells with 7,8-DHF did not result in the expected effect on TrkB phosphorylation and downstream protein activation. Treatment with DMAQ-B1 and CPL503052, CPL503071, and CPL503113 in the differentiated SH-SY5Y model in which TrkB overexpression was induced by retinoic acid confirmed our previous findings observed in SN56 T48 cells. These compounds were able to induce the phosphorylation of TrkB, Akt1, ERK_1/2_, and PLCγ1. However, pretreatment with K252a did not significantly inhibit the phosphorylation of the TrkB receptor (except BDNF), whereas decreased phosphorylation of PLCγ1 and ERK_1/2_ proteins could be observed for BDNF and the low-molecular-weight compounds. The decrease in the Akt1 phosphorylation level after K252a treatment could be observed only for BDNF-treated cells. Moreover, DMAQ-B1 and molecules from the compound library were able to significantly induce the phosphorylation of ERK_1/2_ and Akt1 protein in non-differentiated SH-SH5Y cells (Figure S3).

### 2.4. Evaluation of the Cytoprotective Effect of TrkB Agonists

**Figure 7:**
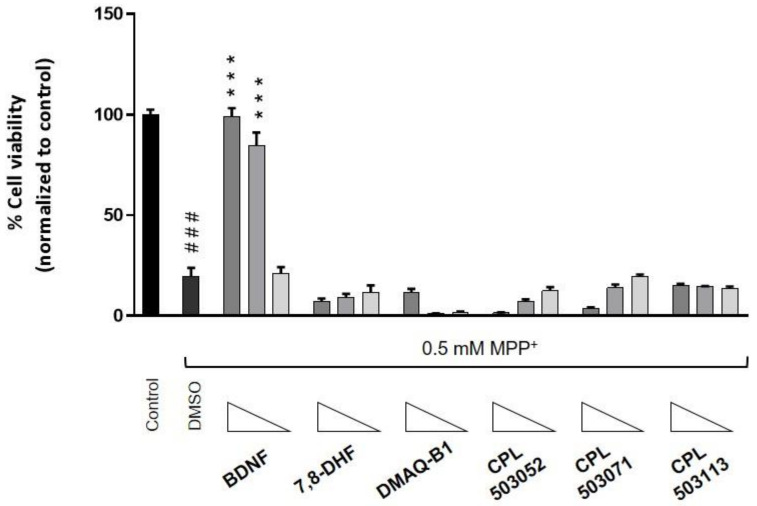
Cytoprotective effect of TrkB agonists in the RA-differentiated SH-SY5Y cell line. Cells were treated with the tested compound for 72 h before cell death was induced by addition of MPP^+^ (0.5 mM). The cytoprotective effect of the compounds was evaluated after cotreating the cells with the compounds and MPP^+^ for another 72 h. Cell viability was monitored using RealTime-Glo™ MT Cell Viability Assay. Bars represent mean ± SEM, *n* = 3. Data were analyzed with one-way ANOVA followed by Dunnett’s post hoc test. *** *p* < 0.001 compared to DMSO-treated group. ### *p* < 0.001 compared to control group.

The cytoprotective effect of BDNF has been demonstrated in many in vitro and in vivo models [28]. Using the neuron-like model, we investigated the cytoprotective effect of reference compounds and molecules selected in previous phases in MPP^+^-induced cytotoxicity. The MPP^+^ was used as an apoptosis inducer in RA-differentiated SH-SY5Y. As shown in Figure 7, BDNF had a dose-dependent cytoprotective effect, which manifested in increased cell viability compared to cells treated with MPP^+^ only. 7,8-DHF, at concentrations of 0.3, 1, and 3 μM, was not able to protect differentiated SH-SY5Y cells against MPP^+^-induced toxicity. Moreover, DMAQ-B1, CPL503051, CPL503071, and CPL503113 did not demonstrate any cytoprotective effect. The remaining reference compounds also did not show any influence on cell viability in this model (Figure S4).

### 2.5. Physicochemical and ADME Characterization

Selected compounds were examined to assess their solubility, chemical stability, and permeability using the PAMPA (parallel artificial membrane permeability assay) and the Caco-2 system, and microsomal stability in mouse and human microsomes. 7,8-DHF was characterized as a soluble compound (303 μM) showing a chemical degradation at pH = 7.4 on a level 15.78% during 4 h. 7,8-DHF presented good permeability in PAMPA (15.8 × 10^−6^ cm/s) and Caco-2 permeability assay (P_app,AB_ = 22.8; P_app,BA_ = 12.42 × 10^−6^ cm/s). Metabolic stability and phase I intrinsic clearance (Cl_int_) were studied using human (HLM) and mouse (MLM) liver microsomes, indicating a moderate degradation rate for 7,8-DHF (HLM Cl_int_ = 52.2; MLM Cl_int_ = 21.3 (μL·min^−1^·mg _protein_^−1^).

The results of physicochemical and ADME analyses performed for the other reference compounds are presented in Table 2. The kinetic solubility analysis (pH = 7.4) indicated low solubility (<100 μM) for 4′-dimethylamino-7,8-DHF, LM22-B20, isocumarin, CPL503052, and CPL503071. Compounds were described as chemically unstable if the degradation was higher than 10% after 4 h. This was the case for the following compounds: 7,8-DHF, DMAQ-B1, CPL503052, CPL503071, and CPL503113; therefore, the last three were excluded from further analysis. Only 4′-dimethylamino-7,8-DHF and NSI-198 showed good permeability (>10^−6^ cm/s) in both PAMPA assay and Caco-2 system. Microsomal stability was assessed in human (HLM) and mouse (MLM) liver microsomes to determine the phase I intrinsic clearance (Cl_int_). In both species, 4′-dimethylamino-7,8-DHF, isocumarin, and NSI-189 showed degradation rates significantly higher than verapamil and were classified as highly unstable; LM22-A4, HIOC, NAS, and OSSK-495385 showed very good metabolic stability, whereas 7,8,3′-THF presented a moderate degradation rate. LM22-B10 was stable in the reaction with MLM and moderately stable in the reaction with HLM. Cl_int_ was not calculated for DMAQ-B1 and GSB-106 due to the instability of this compound in the experimental system and high variability in analytical responses. None of the compounds displayed the expected drug-like profile required for selection as a lead molecule.

### 2.6. Pharmacokinetic/Pharmacodynamic (PK/PD) Profiling of 7,8-DHF

Despite the moderate chemical stability of 7,8-DHF, we decided to reproduce the pharmacokinetic profile and examine the pharmacodynamic profile for 7,8-DHF in BALB/c mice to evaluate the bioavailability and effect on phosphorylation of TrkB and selected downstream proteins (PLCγ1, Akt1, and ERK_1/2_) in vivo. The chemical instability, which was moderate after 4 h, was mitigated by preparing the solution directly before the experiment.

**Figure 8:**
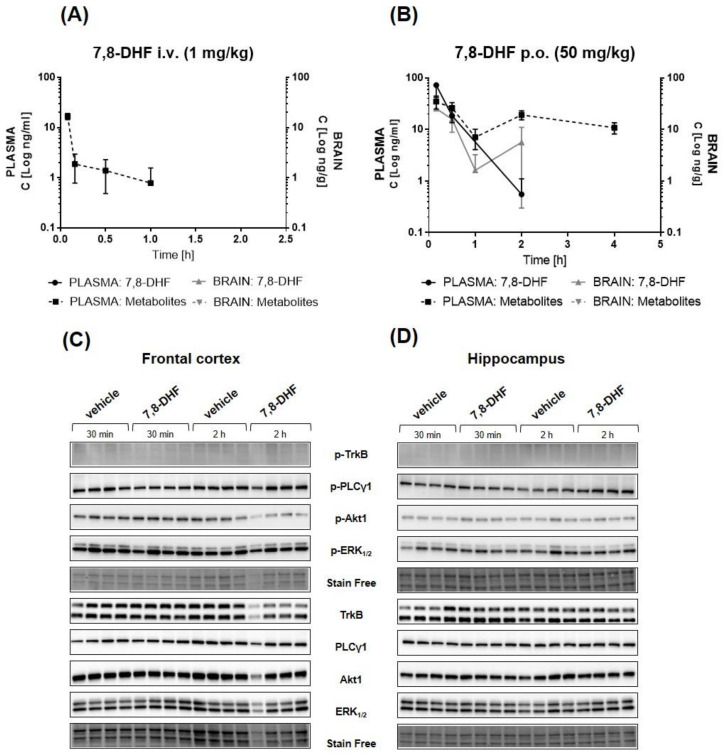
Pharmacokinetic and pharmacodynamic profiles of 7,8-DHF in BALB/c mice. The concentration–time curve of 7,8-DHF and its metabolites (7-hydroxy-8-methoxyflavone and 8-hydroxy-7-methoxyflavone) in plasma and brain lysates after intravenous (**A**) and oral (**B**) administration. Data are expressed as mean ± SEM, *n* = 4. Western blot analysis of the phosphorylation of TrkB and selected downstream proteins in the frontal cortex (**C**) and hippocampus (**D**) after oral administration of 7,8-DHF.

The pharmacokinetic curves for 7,8-DHF in the plasma and the brain after single intravenous (i.v.) (1 mg/kg) and oral (p.o.) (50 mg/kg) administration are presented in Figure 8A,B, respectively. After i.v. injection, 7,8-DHF was not detectable in either plasma or brain samples, suggesting very rapid elimination. After oral administration, 7,8-DHF showed a maximal concentration of 77.43 ng/mL at the first plasma collection time point (10 min post-administration). The concentration decreased in a rapid manner, falling under the detection limit at 1 h post-administration. In the brain, 7,8-DHF was detected at a very low concentration (C_max_ = 6.35 ng/g) after p.o. administration. The calculated PK parameters are presented in Table 3, and detailed results of the analyte concentrations of individual mice are presented in Table S2. Moreover, the concentrations of two major metabolites of 7,8-DHF—7-hydroxy-8-methoxyflavone (7H8M-flavone) and 8-hydroxy-7-methoxyflavone (8H7M-flavone)—were also analyzed [29]. Figure 8A,B presents the concentration determined for the total amount of these two analytes due to bioanalytical limitations related to their identical molecular weights. 7,8-DHF metabolites were detected only in the plasma and peaked 10 min after oral administration and 5 min after i.v. administration, suggesting the rapid transformation of 7,8-DHF and confirming its fast elimination. Furthermore, the impact of 7,8-DHF administration on the activation of TrkB and its dependent pathways in vivo was evaluated by immunoblotting in homogenates from the hippocampus and frontal cortex collected 30 min and 2 h after oral administration. As shown in Figure 8C,D, we did not observe any activation of the TrkB receptor, as measured by receptor phosphorylation (Tyr707) in the analyzed samples. Furthermore, densitometric analysis also indicated no impact of compound administration and metabolites on PLCγ1, Akt1, and ERK1/2 phosphorylation (Figure S5).

### 2.7. Selectivity of 7,8-DHF in a Panel of Various Molecular Targets

Our results obtained for 7,8-DHF using in vitro models with TrkB overexpression, and examination of the pharmacodynamic effects in mouse brain after acute administration, suggest that the 7,8-DHF action observed in vivo (Marco Emili 2020) cannot be explained only by its interaction with the TrkB receptor and its subsequent activation. Therefore, we decided to examine 7,8-DHF at 10 μM in the Eurofins BioPrint^®^ Profile Panel to determine the binding of 7,8-DHF to a broad panel of molecular targets selected and recommended for pharmacological profiling by Bowes et al., 2012 [30].

The binding of 7,8-DHF to 133 molecular targets was investigated using radiolabeled ligands indicating the nature of the interaction. As presented in Table 4, 7,8-DHF was identified as an agonist of adenosine receptor types 1 and 3 (A_1_ and A_3_), melatonin receptor type 3 (MT_3_), and GABAA receptor α1 BZD site (BZD), and an antagonist for adenosine receptor type 2B (A_2B_). 7,8-DHF presented inhibitory activity for non- kinase enzymes such as xanthine oxidase, cyclooxygenase type 2 (COX2), matrix metallopeptidases 2 and 9 (MMP-2 and MMP-9), and Lyn A kinase. For the results of binding studies for all of the 133 examined molecular targets, please see the Supplementary Materials (Table S3).

## 3. Discussion

The BDNF–TrkB pathway is indicated to be an attractive target for the development of novel therapies for central nervous system (CNS) disorders [28]. TrkB is a receptor for neurotrophic factors and might be activated upon binding with BDNF, NT4 (neurotrophin-4) and NT3 (neurotrophin-3) [31]. The binding affinity differs between these molecules, and BDNF and NT4 interact much stronger with TrkB than NT3 [31]. However, the use of the TrkB receptor as a pharmacological target is challenging. The natural ligand BDNF and peptides generally do not cross the blood–brain barrier (BBB); therefore, their systemic administration is not feasible [32]. Registered antidepressants—for instance, imipramine and fluoxetine—are able to allosterically increase BDNF signaling but pose a risk of side effects and the necessity of long-term antidepressant treatment [10,11,33]. Therefore, the search for small molecular TrkB agonists that are able to cross the BBB and mimic BDNF’s action may lead to the development of a novel and effective therapy. 7,8-DHF has been postulated as a TrkB agonist and used as reference molecule for research into the TrkB receptor and development of new agonists [12,13,34]. However, the use of 7,8-DHF as a research tool to study TrkB agonism was questioned by other groups who were not able to confirm TrkB activation [25,26]. In this study, we attempted to verify the pharmacology of 7,8-DHF and other potential reference compounds, and screened a compound library for novel TrkB agonists.

We confirmed that 7,8-DHF binds to the exTrkB domain and determined the binding affinity to be 1.3 μM (Table 1). Jang et al. (2010) and Liu et al. (2014) reported binding affinities of 7,8-DHF in the nanomolar range (15.4 and 12.1 nM, respectively); however, these differences in binding affinity appear to result from the application of different methods because the K_d_ values for BDNF determined by MST were also higher than that of SPR, and were 93 and 1.7 nM, respectively [12,35]. Furthermore, Jang et al. (2010) showed that TrkB dimerizes upon 7,8-DHF binding to the extracellular domain using the pull-down assay [12]. We applied the native electrophoresis method to confirm the ability of 7,8-DHF to dimerize the receptor. However, we were not able to observe a dimerization effect for 7,8-DHF (Figure 4).

It was reported that 7,8-DHF is able to induce TrkB autophosphorylation, which in turn leads to the activation of a downstream cascade and phosphorylation of Akt1 and ERK_1/2_ proteins in the SN56 T48 cell line and primary neurons [12,13]. Our results suggest that the activation of the TrkB receptor by 7,8-DHF in the SN56 T48 cell line does not occur, either in orthosteric or allosteric modes (Figure 3 and Figure S1). Moreover, immunoblotting analysis also confirmed the lack of 7,8-DHF activity on TrkB effector proteins in SN56 T48 and RA-differentiated SH-SY5Y cells (Figure 5 and Figure 6). These results confirm the results obtained by Boltayev et al., (2017) and Todd et al., (2014) in other in vitro cell models—DiscoverX U2OS, PathHunter Assays, and primary neuronal cells—in which no activation of the TrkB receptor was observed [25,26].

To evaluate the functional properties of potential TrkB agonists, we assessed the cytoprotective properties in an induced-toxicity cell model using the differentiated SH-SY5Y cell line. We confirmed BDNF’s protective properties and the activation of downstream signaling related to TrkB (Figure 6 and Figure 7) but were not able to confirm any cytoprotective action of 7,8-DHF. Several groups reported that 7,8-DHF has cytoprotective properties in various in vitro models of induced cytotoxicity: 6-OHDA and MPP^+^ in PC-12 cells, and glutamate in the HT-22 cell line [36,37,38]. In these studies, 7,8-DHF protected cells from apoptosis over a wide range of doses (0.1–25 μM); however, none of these included differentiated SHSY-5Y, in which TrkB is overexpressed (Figure S2). In the mentioned studies, the protective effect of 7,8-DHF was correlated with enhanced HO-1 and SOD activity and JNK signaling inhibition in PC-12 cells, and increased glutathione levels in the HT-22 cell line. This may indicate a cell-specific mode of action, independent of TrkB, for the cytoprotective effect of 7,8-DHF.

To definitively confirm TrkB engagement in the mediation of 7,8-DHF’s effectiveness, a combined pharmacokinetic and pharmacodynamic study in mice was performed. Before the start of the study, physicochemical and ADME properties were assessed. 7,8-DHF is a soluble molecule but tends to degrade at pH = 7.4. 7,8-DHF is permeable according to the PAMPA and in the Caco-2 system and presents a moderate degradation rate in microsomes (Table 3). 7,8-DHF was originally described in the literature as a bioavailable compound that can pass through the BBB and demonstrated a functional effect in a TrkB-dependent manner after acute (5 mg/kg in TrkB F616A knock-in mice) and 14-day treatment (exposed to C57BL/6 in drinking water) [39]. However, further studies in C57BL/6, CD1, and 5xFAD mice indicated unfavorable pharmacokinetic properties for 7,8-DHF. The compound was shown to be orally bioavailable; however, its bioavailability remained low based on low exposure values in relation to the administration of high dosages [29,40]. In our study, BALB/c mice were administered i.v. and p.o. 7,8-DHF. 7,8-DHF was not detectable in the plasma or brain after 1 mg/kg of i.v. administration, suggesting a very rapid elimination process; however, this process was most likely independent of phase I microsome elimination, as evaluated in ADME analysis (Figure 8A, Table 3 and Table 4). 7,8-DHF was detected in the plasma after oral gavage (50 mg/kg), in which it reached a concentration of 72.42 ng/mL. The concentration of 7,8-DHF in the brain was determined to be 10-fold lower than that in the plasma (C_max_ = 6.35 ng/g); however, the compound was not detected in all the brain samples. O-methylated metabolites of 7,8-DHF may also be partially responsible for the pharmacological effects, as they activate the TrkB receptor [29]. 7-hydroxy-8-methoxyflavone and 8-hydroxy-7-methoxyflavone were detected in the plasma, with C_max_ = 34.64 ng/mL, in our study, after oral administration, whereas they were not detected in the brain (Figure 8B, Table 3).

Consecutive pharmacodynamic analysis demonstrated no effect of 7,8-DHF administration on the activation of the TrkB receptor (Tyr706) and phosphorylation of downstream proteins in the hippocampus and frontal cortex collected after 0.5 and 2 h of compound administration (Figure 8C,D). However, it should be noted that, in our study, 7,8-DHF reached a lower concentration in the BALB/c mice’s brains after oral administration than was observed in other studies and mouse strains [29,40]. For this reason, we cannot conclusively state whether the lack of pharmacodynamic effect observed in our study was due either to the compound’s insufficient TrkB activation, or the concentrations of the parental compound and metabolites not being sufficient to induce the TrkB receptor and related signaling in the brain.

Nevertheless, because we did not observe any 7,8-DHF-activated TrkB signaling in vitro, dimerization of the TrkB receptor, or functional effects, we decided to assess the selectivity profile of 7,8-DHF. The BioPrint^®^ Profile Panel showed that 7,8-DHF is able to bind to multiple molecular targets, including A_1_, A_2A_, A_2B_, A_3_, BZD, MT3, and 5-HT2b receptors, and COX2, MMP-2, MPP-9, Lyn A kinase, and xanthine oxidase (Table 4). Most of the listed targets present neuropsychiatric activity. In particular, interaction with A_1A_, A_2A_, BZD, or COX-2 may explain the observed positive behavioral effects of 7,8-DHF [41,42,43]. Compounds targeting the adenosine pathway, depending on the receptor subtype, could be beneficial in several CNS disorders, including epilepsy, pain, and cerebral ischemia. A_1A_ receptors reduce excitatory transmission by the regulation of potassium homeostasis, which leads to the inhibition of glutamate secretion in neuronal cells, whereas the A_2A_ receptor modulates the sensitivity of the dopamine D2 receptor, typically in striatal neurons, which affects motoric functions [44]. The interaction of the GABAA receptor was clinically proved to produce anxiolytic and antidepressant effects via reversing deficits in monoamine transmission in GABAergic machinery [42]. The inhibition of COX-2 with celecoxib, a well-known nonsteroidal anti-inflammatory drug, reversed the behavioral deficits produced by chronic unpredictable stress in rats, and demonstrated efficacy in clinical trials with depressive patients, which was correlated with anti-inflammatory action [43,45,46]. The low selectivity of 7,8-DHF indicates that the pharmacological activity observed in numerous in vivo studies might result from the multitarget action of this molecule. Thus, in light of these results, the current findings should be carefully interpreted because the pharmacological effect of 7,8-DHF might be achieved through interaction with at least several molecular targets.

This study also aimed to re-evaluate the activity of other low-molecular-weight reference compounds reported in the literature as TrkB agonists. We were able to determine the K_d_ values for OSK495385 (2.32 μM), DMAQ-B1 (5.6 μM), and LM22-B10 (83 μM) (Table 1). However, with the exception of DMAQ-B1, we did not observe any orthosteric activity for those compounds or the remaining reference compounds: 7,8,3′-THF, 4′-dimethylamino-7,8-DHF, GSB-106, LM22-A4, HIOC, NAS, isocumarin, and NSI-189 (Figure 3C). These compounds were additionally tested for the positive allosteric modulation of BDNF-TrkB activity but also failed to show activity (Figure S1). The reference compounds induced no TrkB dimerization. Only DMAQ-B1 showed activity in in vitro models activating TrkB effector proteins (Figure 4, Figure 5A–C). However, cotreatment with a tyrosine kinase inhibitor (K252a) did not inhibit DMAQ-B1’s effects, suggesting that the mechanism of action for DMAQ-B1 is independent of TrkB (Figure 6A–C, Figure S3). Furthermore, the reference compounds tested in this study also failed in our functional assessment and did not show any cytoprotective properties (Figure 7). Finally, our physicochemical and ADME profiling indicates unfavorable properties regarding solubility, permeability, and microsomal stability, which also results in poor drug-like features (Table 2). DMAQ-B1 was chemically unstable, thus disqualifying the compound from further studies.

In this study, we also performed screening to select novel, low-molecular-weight TrkB agonists. The library of compounds, initially including 987 molecules, was screened within 17 different chemotypes. Using the MicroScale Thermophoresis technique, 59 molecules were identified as TrkB binders, of which CPL503052, CPL503071, and CPL503113 showed K_d_ values in the range 2.6–44.2 μM (Figure 2, Table 1). We were able to observe TrkB phosphorylation after treatment only with CPL503052, CPL503071, and CPL503113 (Figure 3B). However, we did not observe exTrkB dimerization for this compound in contrast to BDNF (Figure 4); thus, we postulated that the molecule acts via a non-canonical mechanism by activating TrkB monomer signaling [6]. However, further analysis indicated the non-specific activation of downstream proteins after cotreatment with K252, indicating a mode of action that is independent of TrkB for these molecules (Figure 3B, Figure 5B,D and Figure 6A,B, Figure S3). Moreover, these compounds also showed no cytoprotective properties in our study (Figure 7), and further physiochemical profiling indicated poor solubility and chemical instability for CPL503052, CPL503071, and CPL503113, which may affect the compounds’ activity. Due to the unfavorable physicochemical properties of CPL503052, CPL503071, and CPL503113 (Table 2) and nonspecific activation of downstream signaling, these molecules were excluded from further development. To summarize, none of the chemotypes tested in this study were proven to be active or to possess drug-like properties, as assessed in our drug-discovery approach.

Thus, the demonstrated results corroborate the notion that TrkB is not an easily “druggable” target for the discovery of orthosteric agonists. Indeed, the activity of the 7,8-DHF was tested in many preclinical studies. However to this date, no clinical trials investigating 7,8-DHF pharmacological activity in human subjects were reported (based on www.clinicaltrials.gov accessed on 14 July 2021, no matches can be found after searching for following records: “7,8-DHF”, “7,8-dihydroxyflavone”). Our study suggests that 7,8-DHF’s pharmacology is more complex than initially assumed. Despite the comprehensive analyses carried out in this study, it remains difficult to clearly identify the mechanisms by which 7,8-DHF achieved the biological effects described in numerous in vivo studies. Our results suggest that the activity of 7,8-DHF in vivo is not mediated through direct TrkB activation but can be attributed to other molecular targets, potentially activated by the molecule or also by the molecule’s derivatives. Based on the latest findings, allosteric interaction with TrkB offers new opportunities, and a shift towards the crossed transmembrane domain of the TrkB receptor as a target for novel agonist pharmacophores should be considered in future development engaging the BDNF/TrkB signaling pathway [33]. Therefore, 7,8-DHF should be carefully considered as a reference compound for a TrkB receptor agonist and BDNF-mimicking agent in further analysis.

## 4. Materials and Methods

### 4.1. Molecules Tested for Trkb Agonism

The reference compounds were purchased from different sources: DMAQ-B1, LM22A-4, LM22B-10, and HIOC were from Tocris (Bristol, UK). 7,8-DHF and NSI-189 were from Fluorochem (TriMen Chemicals, Lodz, Poland), OSSK-495385 from Mcule (Palo Alto, CA, USA), and NAS was purchased from TCI (Argenta, Poznan, Poland). Compounds including 7,8,3′-THF, 4′-dimethylamino-7,8-DHF, GSB-106, isocumarin, and 7,8-DHF metabolites (7H8M-flavone and 8H7M-flavone) were synthetized in house by CelonPharma’s Medicinal Chemistry department. The compound library was purchased from Enamine (Riga, Latvia). The CPL503052 (didemethylasterriquinone D, MW = 370.36 g/mol) [47], CPL503071 (MW = 438.48 g/mol) [47], and CPL503113 (MW = 323.38 g/mol) [48] were from the internal compound library of Celon Pharma (Kazun Nowy, Poland). The chemical structures of the molecules are presented in Table S4: Chemical structure of compounds. The chemical compounds were dissolved in DMSO. BDNF was delivered by MilliporeSigma (Saint Louis, MO, USA).

### 4.2. Primary Screening and Analysis of Compound Interactions with exTrkb

#### 4.2.1. Preparation of exTrkB Protein

The gene encoding the extracellular domain of the human TrkB receptor (exTrkB), residues 1−430, was cloned into the pCMV2 expression vector. Protein expression was performed in suspension-cultivated mammalian ExpiCHO-S cells using the ExpiFectamine (Thermo Scientific, Thermo Fisher Scientific, Waltham, MA, USA) transfection reagent. The secreted protein was purified using a four-step protocol. Clarified culture medium was precipitated with ammonium sulfate added to a final concentration of 1.5 M and centrifuged (235,000× *g*, 60 min at 4 °C). The supernatant after centrifugation was applied on a HiTrap Phenyl column (GE Healthcare, Chicago, IL, USA) equilibrated with buffer A (25 mM Tris, 1 mM DTT; pH 7.5) with 1.5 M ammonium sulfate. Bound proteins were eluted with a linear gradient of buffer A. The eluted proteins were dialyzed overnight in buffer A and loaded on a CaptoQ ImpRes column (GE Healthcare) equilibrated with buffer A with 50 mM NaCl. The bound proteins were eluted with a linear gradient of buffer A with 1 M NaCl. The fractions containing exTrkB protein were pooled, and MnCl_2_ and CaCl_2_ (1mM final concentration each) were added. The sample was purified on a HiTrap Con A 4B column (GE Healthcare) equilibrated with buffer B (25 mM Tris, 0.5 M NaCl, 1 mM MnCl_2_, 1 mM CaCl_2_; pH 7.5) and eluted with buffer C (25 mM Tris, 0.1 M NaCl, 0.4 M α-d-glucopyranoside; pH 6.5). To improve the protein recovery, buffer C flow was paused nine times during elution. The eluted fractions were concentrated and purified by size-exclusion chromatography on a HiLoad 16/600 Superdex 200 column (GE Healthcare) in buffer A with 0.5 M NaCl. The protein samples were concentrated to 1 mg/mL, and buffer was exchanged (PBS; pH 7.4). Samples were frozen in liquid nitrogen and stored at −80 °C. The protein purity was estimated by SDS–PAGE followed by Coomassie Brilliant Blue staining. The protein concentration was determined from the absorption at 280 nm using an UV NanoDrop 1000 spectrophotometer (Thermo Scientific).

The exTrkB was labeled with a Monolith Protein Labeling Kit RED-NHS 2nd Generation (NanoTemper Technologies GmbH, Munich, Germany) according to the manufacturer’s protocol. The labelling efficiency was always determined spectrophotometrically with NanoPhotometer^®^ NP80 (Impel) in line with the manufacturer’s recommendations. Labelled exTrkB protein was aliquoted and stored at −80 °C to avoid repeated freeze–thaw cycles. Before analysis, the exTrkB protein was thawed on ice and diluted with MST buffer (50 mM Tris pH = 8; 150 mM NaCl; 10 mM MgCl_2_; 0.1% Tween20) to the desired concentration.

#### 4.2.2. Single-Point and Binding Affinity Screening with Microscale Thermophoresis (MST)

For primary screening (single-point MST experiments for signal amplitude determination), each of the 987 screened compounds were diluted in MST buffer + 0.1% Tween20 to a final concentration of 0.5 mM. The tested compounds were mixed with exTrkB solution (the final concentration of exTrkB was 20 nM) and centrifuged (10,000× *g*, 4 min at 4 °C) to eliminate protein and compound aggregates. Before loading to capillaries (Monolith NT.115 Capillaries, NanoTemper Technologies GmbH), the samples were incubated for 5 min at 25 °C and then placed into the MST apparatus (Monolith, NanoTemper Technologies GmbH). The experiments were performed using nano-blue excitation, LED light adjusted to 20% excitation power, and MST power set to medium, and the fluorescence signal during thermophoresis was detected after 30 s. Single-point MST experiments were performed by the determination of the amplitude signal between the unbound and bound state between the molecule and exTrkB, which was analyzed using the MO.Control Software version 1.6 (NanoTemper Technologies GmbH). A reference sample containing only exTrkB and the equivalent DMSO concentration (*v*/*v*) was included in each individual MST experiment. At least three technical replicates were prepared for each of the tested compounds. A binding affinity study was performed for 113 of the selected compounds at 16 concentration points. In this step, the compound solubility was visually verified for insoluble particles or turbidity. Compound concentration ranges were adjusted individually for each tested compound. The samples were processed under the same regime and experimental conditions as described above. Binding affinity screening (the determination of dissociation constants, K_d_) was performed with the MO Affinity Analysis Software version 2.3 (NanoTemper Technologies GmbH) using the K_d_ binding model.

#### 4.2.3. Ligand-Induced exTrkB Dimerization Assay

The native exTrkB, BDNF, and tested compounds were separately diluted with PBS (pH 7.4) to a final concentration of 2.5 μM. Then, exTrkB, BDNF, or the tested compound were mixed (1:1 *v*/*v*) and incubated at 25 °C for 10 min, and 2X Sample Buffer (0.6 M Tris, 20% glycerol, 0.02% Bromophenol Blue, pH 6.8) was added to the samples. Afterwards, the samples were loaded into Stain-Free gels (10% Mini-PROTEAN^®^ TGX Stain-Free™ Protein Gels; Bio-Rad, Hercules, CA, USA) and separated in non-reducing conditions using SDS-free electrophoresis buffer (192 mM glycine, 25 mM Tris; pH 8.3). Visualization of the protein bands was performed using a ChemiDoc MP Imaging System (Bio-Rad).

### 4.3. In Vitro Functional Experiments

#### 4.3.1. Cell Lines and Materials

The SN56 T48 cell line with stable overexpression of the TrkB receptor was purchased from Emory University (Atlanta, GA, USA) and cultivated according to the protocol described in Jang et al., 2010 [12]. The cell lines were cultivated with the following reagents: DMEM High Glucose, fetal bovine serum (FBS), Penicillin-Streptomycin Solution 100×, and Trypsin-EDTA 1×; all were purchased from Biowest, and G418 was from ThermoFisher Scientific. For the ELFI experiments, cells were seeded (8000/well) on a PDL-treated 96-well plate (BioCoat™, Corning Inc., Corning, NY, USA) and 6-well plate (400,000/well) for lysate collection and immunoblot analysis.

The SH-SY5Y cell line was purchased from MilliporeSigma and cultured in growth medium: MEM (w/Earle’s Salts; w/l-Glutamine) + Ham’s F12 (w/l-Glutamine) (1:1 *v*/*v*), 1 × Antibiotic-Antimycotic, 1 × MEM Non-Essential Amino Acids (all reagents from Biowest, Riverside, MO, USA) and 15% FBS (Gibco, Thermo Fisher Scientific, Waltham, MA, USA). Cells were passaged using 0.25% Trypsin-EDTA (Biowest) and cultured in standard conditions (5% CO_2_, 37 °C with 95% relative humidity).

To differentiate SH-SY5Y, cells were seeded in a cell culture flask at a density of 10,000 cells/cm^2^ in standard growth medium containing 10 μM retinoic acid (RA; MilliporeSigma) and cultured for 7 consecutive days. Then, differentiated cells were harvested by trypsinization (Trypsin-EDTA 1×, 3 min at 37 °C), centrifuged (250× *g*; 5 min at 25 °C) and counted. The desired experiment cells were seeded in differentiation medium (MEM:Ham’s F12 growth medium +10 μM RA) in multi-well plates at the following densities: 1,000,000 cells/well on a 6-well plate for lysate collection and immunoblot analysis, and 12,000 cells/well into a PDL-treated 96-well plate (BioCoat™, Corning Inc.) for neuroprotection experiments.

#### 4.3.2. Enzyme-Linked Fixed Cell Immunoassay (ELFI) Method for Allosteric and Orthosteric Examination

In the orthosteric agonist mode experiments, starved cells (5 h) were treated with compounds (0.125–100 μM; volume, 100 μL/well) or BDNF (0.01–10 nM; volume, 100 μL/well) dissolved in culture medium. In the allosteric modulation mode experiments, starved cells (5 h) were treated with final compound concentrations of 3, 10, and 30 μM; volume, 50 μL/well) in the presence of a rising concentration of BDNF (final concentration in well, 0.15–5 nM; volume, 50 μL/well). Corresponding treatment with DMSO (*v*/*v*) was performed for a control. Cells were treated for 15 min in both the orthosteric and allosteric modulation mode experiments.

The analysis of TrkB receptor activation was performed using the ELFI method adapted from Boltayev et al. (2017) with some modifications [26]. After compound treatment, experiments were stopped by fixing cells with 10% buffered formalin (Kolchem, Lodz, Poland) for 20 min (volume, 100 μL/well). Afterwards, cells were permeabilized with washing buffer (TBS+ 0.1% Tween20) for 15 min, and endogenous peroxidases were quenched by 20 min of incubation with 1% H_2_O_2_ dissolved in TBS (each solution: volume, 100 μL/well). Epitopes were blocked with 10% BSA (Biowest) dissolved in washing buffer for 2 h (volume, 100 μL/well) and incubated with anti-pTrkB (Tyr707) primary antibody (Cell Signaling Technology, Danvers, MA, USA) diluted in washing buffer (1:500; overnight at 4 °C; volume, 100 μL/well). After repeated washing, cells were incubated with HRP-linked anti-rabbit antibody (Cell Signaling Technology) for 2 h (1:1000; RT; volume, 100 μL/well) and washed. The cells were then incubated with 100 μL of ImmunoCruz™ Western Blotting Luminol (Santa Cruz Biotechnology, Dallas, TX, USA) for 1 min in the dark, and the signals were measured using a microplate luminometer (VICTOR Light 1420-060, PerkinElmer^®^, Waltham, MA, USA). For non-specific binding, cells were treated, with the exception of primary antibody.

#### 4.3.3. Downstream Signaling Analysis

On the day of the experiments, cells were starved for 5 h with appropriate culture medium. SN56 T48 cells were treated with reference compounds at 100 μM or 1, 10, or 100 μM concentrations of the selected molecules, and 1, 10, and 100 ng/mL of BDNF. Differentiated SH-SY5Y cells were treated with 1, 10, or 100 μM concentrations of the tested molecules and 1, 10, and 100 ng/mL of BDNF for 15 min. For compound specificity, the evaluation cell lines were first pretreated with 1 μM K252a (MilliporeSigma, Saint Louis, MO, USA) for 15 min and then cotreated with 100 μM concentrations of the tested molecules.

Compound treatment was stopped by lysing cells with N-PER™ Neuronal Protein Extraction Reagent (Thermo Fisher Scientific) containing 1 × PhosSTOP™ (Roche, Basel, Switzerland), 1 × Halt Protease Inhibitor Single-Use Cocktail (ThermoFisher Scientific) and 5 mM EDTA. The lysates were collected by scraping, and centrifuged (8000× *g*; 10 min at 4 °C), and the protein concentration was determined using a BCA Pierce kit (ThermoFisher Scientific). The lysates were stored at −80 °C until further processing. Samples containing 20 μg of protein were further denatured in 4 × Laemli Buffer with 5% β-mercaptoethanol at 95 °C for 5 min and electrophoresed on gels prepared using a 10% TGX Stain-Free™ FastCast™ Acrylamide Kit (Bio-Rad). Afterwards, the proteins were transferred (wet transfer, 100 V, 60 min) onto nitrocellulose membranes (GE Healthcare, Chicago, IL, USA), which were blocked in 5% BSA in TBS-T (TBS + 0.1% Tween20) for 2 h. The blots were then probed (4 °C; overnight) with the following primary antibodies: phospho-TrkB (Tyr706/707) (1:1000), TrkB (80E3) (1:1000), phospho-PLCγ1 (Tyr783) (1:1000), PLCγ1 (1:1000), phospho-ERK1/2 (Thr202/Tyr204) (D13.14.4E) (1:4000), ERK1/2 (1:3000), phospho-Akt (Ser473) (D9E) (1:4000), and Akt (1:3000); all the antibodies were purchased from Cell Signaling Technology. After repeated washes (TBS-T), the proteins were detected with HRP-conjugated anti-rabbit antibodies (1:4000, RT, 2 h) (Cell Signaling Technology), and the blots were incubated with Clarity Max ECL Western Blotting Substrate (Bio-Rad). Blot images and chemiluminescence signals were captured using a ChemiDoc MP Imaging System, and densitometry values were calculated using the Image Lab software (Bio-Rad). The experiments were performed with three biologically independent replicates, and the chemiluminescence signals of each protein were normalized to the total protein volume bands visualized in the Stain-Free gels.

#### 4.3.4. Evaluation of Neuroprotective Effect of TrkB Agonists

The experiment design was adopted from the article published by Presgraves et al., (2004), with the following modifications [49]. Differentiated SH-SY5Y were prepared as described in Section 4.3.1. On the day of the experiment (Day 0), culture medium containing 10 μM RA was changed for treatment medium composed of MEM:HAM’S F12 (1:1 *v*/*v*), 1 × Antibiotic-Antimycotic, and 1 × MEM Non-Essential Amino Acids, with the FBS reduced to 0.5%. In this step, RealTime-Glo™ reagents (Promega, Madison, WI, USA) were added to the treatment medium (to a final concentration of 0.5 × ). Cell viability was measured after 1 h of incubation at 37 °C using a luminometer (VICTOR Light 1420-060) for normalization. Then, the tested compounds and BDNF were diluted in culturing medium as 2 × concentrate (final concentrations of 0.3, 1, and 3 μM for the small molecular compounds and 1, 10, and 100 ng/mL for BDNF) and added into wells for 72 h of incubation (volume, 100 μL/well). Then (Day 3), the treatment medium was replaced with fresh medium containing 0.5 × RealTime-Glo™ reagents (volume, 100 μL/well), and the cell viability was measured for normalization. Then, compounds and BDNF were added as 4 × concentrates (volume, 50 μL/well). In addition, MPP^+^ was also diluted in treatment medium and added to the wells as a 4 × concentrate (final concentration, 0.5 mM; volume, 50 μL/well). The cells were incubated for a further 72 h at 37 °C. On Day 6, the cell viability was measured 1 h after replacing the medium with the medium containing 0.5 × RealTime-Glo™ (volume 100 μL/well). The experiments were performed with three biological independent replicates, and the final cell viability (endpoint, Day 6) was normalized to the normalized cell viability calculated on Day 3.

### 4.4. Physiochemical Characterization and ADME Profiling

#### 4.4.1. Chemical Stability

The kinetic solubility was determined by the shake-flask protocol [50,51]. Incubation of the appropriate compounds (500 μM) was performed in aqueous buffers (0.1 M phosphate-buffered saline, pH 7.4) at 25 °C with stirring at 500 rpm. The samples for the determination of chemical stability were taken at the initial time and after 4 h of incubation and diluted with 1 volume of acetonitrile. The samples for the determination of kinetic solubility were taken after 4 h of incubation, filtered through 0.22 μm filters, and diluted with 1 volume of acetonitrile. The sample concentration was determined by UHPLC–UV/Vis (Agilent Technologies, Santa Clara, CA, USA). A calibration curve was prepared to quantify the contents of the compound in the test solution.

#### 4.4.2. Parallel Artificial Membrane Permeability Assay (PAMPA)

An artificial membrane was prepared in 96-well filter plates (Millipore MultiScreen IP Filter Plate). Each well of the donor plate was coated with 5 μL of the 2% lecithin/dodecane solution. Next, in each well of the donor plate, 150 μL of a compound-containing donor solution (compound dissolved in 5% DMSO, PBS) was added. All the compounds were added at a concentration of 10 μM. The compounds were prepared from 10 mM stock solutions in DMSO. The 300 μL of buffer (5% DMSO in PBS, pH 7.4) was transferred to each well of the PTFE acceptor plate in technical triplicates. The compound-filled donor plate was placed into the acceptor plate. Incubation was carried out for 4 h at room temperature.

Samples were taken after incubation from donor and acceptor compartments, diluted with 2 volumes of acetonitrile containing a 200 nM concentration of the internal standard (imipramine). The solutions from the donor plate were diluted if necessary. Testosterone (10 μM) and atenolol (10 μM) were used as high and low reference permeability compounds, respectively.

The compound concentration was determined by LC-MS/MS (an Agilent 6460 MS/MS mass spectrometer equipped with Agilent Infinity II 1290 UHPLC, Agilent Technologies). A calibration curve was prepared to quantify the contents of the compound in the test solution.

#### 4.4.3. Caco-2 Permeability

Passive permeability was investigated in 24-well-plate format using differentiated Caco-2 cells (CacoReady 24 well, ReadyCell, Barcelona, Spain), according to a previously published protocol [52] with proprietary modifications and with restrictions described in the product manual. Bidirectional (apical-to-basal and basal-to-apical, AB, and BA) passive permeability was assessed in HBSS buffer containing calcium, magnesium, 5 mM glucose, and a modified transporter inhibitor cocktail (final concentrations of 5 μM elacridar and 20 μM cyclosporine A). Samples were collected at time 0 (from the donor compartment) and after 2 h (from the donor and the acceptor compartments) and diluted with the assay buffer (donor samples) and MeOH (all samples) containing internal standard (100 nM imipramine), followed by a brief centrifugation to remove any buffer precipitates prior to LC-MS analysis. Compounds’ contents in the samples were measured using a UHPLC system (Vanquish Flex, Thermo Fisher Scientific) coupled to a Triple Quad MS detector (QTRAP 5500+, Sciex, Framingham, MA, USA) in multiple reaction monitoring (MRM) mode. Conditions of gradient LC separation were adjusted individually to each compound. Passive permeability (P_app_) was then calculated according to the product manual. Atenolol and diclofenac were used as low and high permeability reference compounds, respectively. Additionally, the pre-assay control of transepithelial electric resistance (TEER) and post-assay control of lucifer yellow (LY) flux across the monolayer were performed for each well to assess the integrity of the monolayer.

#### 4.4.4. Metabolic Stability

First-phase metabolic stability was investigated in vitro using human (HLM) and mouse (MLM) liver microsomes (Gibco, Thermo Fisher Scientific) as described in Gunerka et al., 2020, with some modifications [53]. Verapamil and warfarin were used as high and low clearance reference compounds, respectively. Test compounds (1 μM) were incubated in the dark at 37 °C with mixing (500 rpm) in 100 mM potassium phosphate buffer containing microsomes (0.5 mg/mL), NADPH (1–1.2 mM), and MgCl_2_ (3.3 mM). NADPH, a cofactor for CYP family enzymes, was prepared prior to the experiment by reducing NADP with G6P dehydrogenase (Merck, Darmstadt, Germany) (13.2 mM G6P, 5.2 mM NADP, 3.2 U/mL G6P dehydrogenase, 25 min at 30 °C, 500 rpm). The negative control contained buffer instead of NADPH solution. Samples were collected at 0, 10, 20, and 40 min or 0 and 40 min for the negative controls. The reaction was stopped by protein precipitation in 4 volumes of ice cold MeOH containing 100 nM imipramine as an internal standard. Then, the extract was mixed (1 min, 1000 rpm) and centrifuged (2250× *g*, 20 min, 4 °C), and the supernatant was transferred to an analytical plate and further diluted with water for LC-MS analysis. The compounds’ disappearance from the reactions was measured using the LC-MS system as described for Caco-2 permeability. The elimination rate constant (k) was calculated as a module slope of a log-linear regression of substrate concentration vs. time. Then, the intrinsic clearance (Cl_int_) was calculated as k per microsomal protein content (d = 0.5 mg_protein_/mL).

#### 4.4.5. In Vivo

The PK/PD profiling of 7,8-DHF was performed in BALB/c male mice weighing 20–25 g. The animals were obtained from the Center of Experimental Medicine (University of Bialystok, Bialystok, Poland) and housed under standard laboratory conditions. Mice had unlimited access to water and food. All the procedures were conducted according to the guidelines of the local ethical commission (appl no. 06/2019, perm. no 10/2019). The animals were subjected to 7,8-DHF at a dose of 50 mg/kg (5% DMSO, 1.5% methylcellulose in water) per os (p.o.) and 1 mg/kg (30% PEG 400 in 0.9% NaCl) for intravenous (i.v.) administration. Four animals per time point were sacrificed at 10 min, 30 min, 1 h, 2 h, and 4 h after per os administration and 5 min, 10 min, 30 min, 1 h, and 2 h after i.v. administration. The blood (0.5 mL) was collected by heart puncture using probes with anticoagulants (K2EDTA). Plasma samples were obtained by blood centrifugation (15 min, 3000× *g* at 23 °C). The whole brain was dissected from the skull and washed with cold 0.9% NaCl on ice. The left hemisphere was quickly frozen in liquid nitrogen and stored at −80 °C for further bioanalytical analysis. From the right hemisphere, the hippocampus and frontal cortex were isolated, weighed, and stored at −80 °C after freezing in liquid nitrogen. Isolated structures were homogenized in N-PER cocktail (10 μL for every 1 mg of tissue) using a hand homogenizer, and then processed as described in Section 4.3.3.

For bioanalytical evaluation, brain samples were homogenized in water (3 mL for every 1 g of brain tissue). Plasma and brain samples were then prepared by precipitating proteins and extracting analytes using acetonitrile with the addition of the internal standard: 150 μL of extraction solution was added to 50 μL of biological material, mixed for 1 min and centrifuged (12,900 RPM, 4 min, 4 °C), and the supernatant was collected for analysis. Standard solutions including calibration standards and quality control samples were prepared by spiking 45 μL of biological material with 5 μL of standard working solution, adding 150 μL of extraction solution, and then mixing, centrifuging, and collecting the supernatant for analysis. The concentrations of analytes in the plasma and brain samples were determined using a UPLC-MS/MS instrument (Agilent Technologies) and chromatographic columns (Agilent Zorbax Eclipse 50 × 2.1 mm; 1.7 μm).

#### 4.4.6. Binding Study

7,8-DHF was analyzed for binding in a broad panel of molecular targets recommended by Bowes et al., 2012 [30]. The BioPrint^®^ Profile Panel service offered by Eurofins Discovery (Poitiers, France) was chosen to perform selectivity analysis.

#### 4.4.7. Statistics

Statistical analysis and graphical presentation of the obtained results were conducted using the GraphPad Prism software (version 7). The statistical significance of differences between the means of the treated groups and the mean of the control group was determined using one-way ANOVA followed by a Dunnett post hoc comparison. A *p*-value < 0.05 was considered statistically significant.

## Figures and Tables

**Table 1:** Summary of results obtained for compounds described in the literature as TrkB agonists and molecules identified as TrkB orthosteric agonists during the screening phase.

Compound	K_d_ [μM]	EC_50_ [μM]	PAM Activity	TrkB Dimerization
BDNF	0.093	0.004	NO	YES
7,8-DHF	1.3	0	NO	NO
OSK495385	2.32	0	NO	NO
DMAQ-B1	5.6	5	NO	NO
LM22-B10	83	0	NO	NO
7,8,3′-THF	0	0	NO	NO
4′-dimethylamino-7,8-DHF	0	0	NO	NO
GSB-106	0	0	NO	NO
LM22-A4	0	0	NO	NO
HIOC	0	0	NO	NO
NAS	0	0	NO	NO
Isocumarin	0	0	NO	NO
NSI-189	0	0	NO	NO
CPL503071	25	6.6	NO	NO
CPL503113	44.2	37	NO	NO
CPL503052	2.6	51	NO	NO

**Table 2:** Results of performed physiochemical and ADME analyses. Kinetic solubility was determined in terms of μM concentration. Chemical stability data (pH = 7.4) are presented as % of compound degradation of initial compound concentration in the analyzed sample. Results of parallel artificial membrane permeability assay (PAMPA) are expressed as 10^−6^ cm/s of compound permeability through biomembrane. Results of passive permeability through the Caco-2 monolayer are given as apparent permeability (P_app_) from apical to basal compartment (AB) or in the opposite direction (BA). Metabolic stability is given as intrinsic clearance (Cl_int_) in human (HLM) and mouse (MLM) liver microsomes per time per amount of microsomal protein.

Compound	Kinetic Solubility [μM]	Chemical Stability [% of Degradation]	PAMPA [10^−6^·cm/s]	P_app,AB_ [10^−6^·cm/s]	P_app,BA_ [10^−6^·cm/s]	Cl_int_ HLM [μL·min^−1^ ·mg_protein_^−1^]	Cl_int_ MLM [μL·min^−1^ ·mg_protein_^−1^]
7,8,3′-THF	168	Stable	1.1	3.00	11.14	49.4	26.6
4′-dimethylamino-7,8-DHF	46.6	1.31	15.5	15.61	9.03	175.7	365.1
DMAQ-B1	500	12.76	6.93	0.00	0.00	**	**
GSB-106	152.7	Stable	0.85	**	**	**	**
LM22-A4	397	Stable	0.01	0.00	0.12	3.7	0.0
LM22-B10	<1	4.18	2.36	6.47	4.97	29	4.8
HIOC	437	Stable	1.24	1.11	2.29	0.0	2.1
NAS	404	Stable	<0.01	8.86	8.00	0.3	1.5
Isocumarin	5	Stable	0.22	1.60	2.18	780.7	407.4
OSSK-495385	500	Stable	0.03	0.61	10.86	10	3.3
NSI-189	500	0.65	8.91	28.23	13.22	466.6	1316.7
CPL503052	21	89.84	*	*	*	*	*
CPL503071	67,8	38.67	*	*	*	*	*
CPL503113	195	12.43	*	*	*	*	*

* Not analyzed. ** Unstable in the experimental system.

**Table 3:** Pharmacokinetic parameters determined for 7,8-DHF and its metabolites in mouse brain and plasma after oral (p.o.) and intravenous (i.v.) administration.

		7,8-DHF p.o. (50 mg/kg)			7,8-DHF i.v. (1 mg/kg)		
		AUC	C_max_	T_max_ [h]	AUC	C_max_	T_max_ [h]
PLASMA	7,8-DHF	20.9	72.42 ng/mL	0.16	N/D	N/D	N/D
	7H8M-flavone 8H7M-flavone	61.8	34.68 ng/mL	0.16	2.2	16.72 ng/mL	0.08
BRAIN	7,8-DHF	5.2	6.35 ng/g	0.16	N/D	N/D	N/D
	7H8M-flavone 8H7M-flavone	N/D	N/D	N/D	N/D	N/D	N/D

N/D—not determined.

**Table 4:** Binding of 7,8-DHF to proteins assessed in BioPrint^®^ Profile Panel. The effect of 7,8-DHF at a concentration of 10 μM was examined against members of several protein families, including G Protein-Coupled Receptors (GPCRs), transporters, ion channels, nuclear receptors, kinases, and non-kinase enzymes. The table presents results showing an inhibition of control specific binding higher than 50%.

Target	Assay Type	Family	% Inhibition of Control Specific Binding
A_1_	Agonist	Receptor	99
Xanthine oxidase/ superoxide O2-scavenging		Non-Kinase Enzyme	91.5
MT_3_ (ML_2_)	Agonist	Receptor	86
COX2		Non-Kinase Enzyme	81.6
A_2B_	Antagonist	Receptor	78.3
A_2A_	Agonist	Receptor	74.9
MMP-9		Non-Kinase Enzyme	72
BZD	Agonist	Ion Channel	70.6
A_3_	Agonist	Receptor	68.2
MMP-2		Non-Kinase Enzyme	56.5
Lyn A kinase		Kinase	52.6
5-HT_2B_	Agonist	Receptor	50.2

## Data Availability

The data presented in this study are available in this article and related Supplementary Materials.

## Supplementary Materials

The following are available online at https://www.mdpi.com/article/10.3390/ph14080704/s1. Figure S1. Results for compounds’ activity tested in PAM mode, Figure S2. TrkB expression in RA-differentiated SH-SY5Y cell line, Figure S3. Effect of compound treatment in naïve SH-SY5Y cell line, Figure S4. Effect of reference compounds on cytoprotection, Figure S5. Densitometry analysis of protein phosphorylation in PD analysis, Table S1. K_d_ values determined for screened compounds by MST technique, Table S2. Detailed results of analytes’ concentrations in PK analysis, Table S3. BioPrint^®^ Profile Panel results for 7,8-DHF, Table S4. Chemical structure of compounds.
