# Supplementary material for: Do Small Molecules Activate the TrkB Receptor in the Same Manner as BDNF? Limitations of Published TrkB Low Molecular Agonists and Screening for Novel TrkB Orthosteric Agonists
Source: Pharmaceuticals (Basel). 2021 Jul 21;14(8):704. doi: 10.3390/ph14080704 (PMC8398766; doi:10.3390/ph14080704)
Supplement: Supplementary file 1 [file pharmaceuticals-14-00704-s001.zip › Supplement.pdf]

Table S1. K_d values determined for screened compounds by MST technique.

Compound	K_d [uM]	Compound	K_d [uM]	Compound	K_d [uM]
**15770915**	658	**15771895**	58	**503-049-01**	0
**15770764**	540	**503-143-06**	55	**15770749**	0
**15770789**	530	**503-073-01**	54,7	**503-110-02**	0
**15772279**	487	**15772074**	54	**503-054-02**	0
**15772161**	454	**503-071-08 (MIX)**	49,7	**503-039-01**	0
**15770738**	450	**15771938**	45	**503-146-07**	0
**15772084**	437	**503-113-01**	44,2	**503-044-11**	0
**15770816**	409	**15772026**	40	**15772288**	0
**15770643**	408	**15772201**	28,7	**15770641**	0
**15772332**	384	**503-071-08**	25	**503-013-01**	0
**15770782**	336	**15772135**	21,8	**503-048-01**	0
**503-125-03**	305	**15772089**	19,7	**15770807**	0
**15772096**	278	**15772141**	15,7	**LM22-A4**	0
**15770817**	268	**15770805**	12,6	**503-119-01**	0
**15770808**	254	**15772080**	10,4	**503-070-01**	0
**15772002**	226	**DMAQ-B1**	5,6	**HIOC**	0
**15770916**	207	**15772115**	5,4	**15770832**	0
**15770892**	194	**15772254**	5,3	**15770835**	0
**15770722**	189	**15772271**	4,81	**15770806**	0
**15770644**	164	**503-104-02**	4,235	**NSI-189**	0
**15772674**	149	**503-112-01**	3,8	**503-125-04**	0
**503-144-01**	148	**503-052-03**	2,6	**15772780**	0
**15771979**	137	**OSK495385**	2,32	**15770845**	0
**503-132-07-04**	137	**503-072-06**	1,6	**GSB-106**	0
**15770706**	136	**7,8-DHF**	1,3	**503-133-01**	0
**15770919**	128	**15772293**	0,512	**503-091-01**	0
**15772181**	128	**15772236**	0,054	**503-023-02**	0
**15770778**	119	**7,8,3′-THF**	0	**15772131**	0
**15772812**	109	**503-109-01**	0	**15770879**	0
**503-021-01**	97	**503-025-02**	0	**4′-N,N-dimetylo-7,8-DHF**	0
**503-126-03**	95	**503-012-05**	0	**503-088-01**	0
**15772123**	94	**503-034-02**	0	**15770867**	0
**15772132**	94	**503-124-01**	0	**503-015-03**	0
**15772248**	94	**503-144-01**	0	**15770639**	0
**LM22-B10**	83	**15772188**	0	**503-016-04**	0
**15772068**	78,9	**503-089-01**	0	**Isocumarin**	0
**15772138**	72,1	**503-140-01**	0	**15770827**	0
**15770812**	60	**NAS**	0		

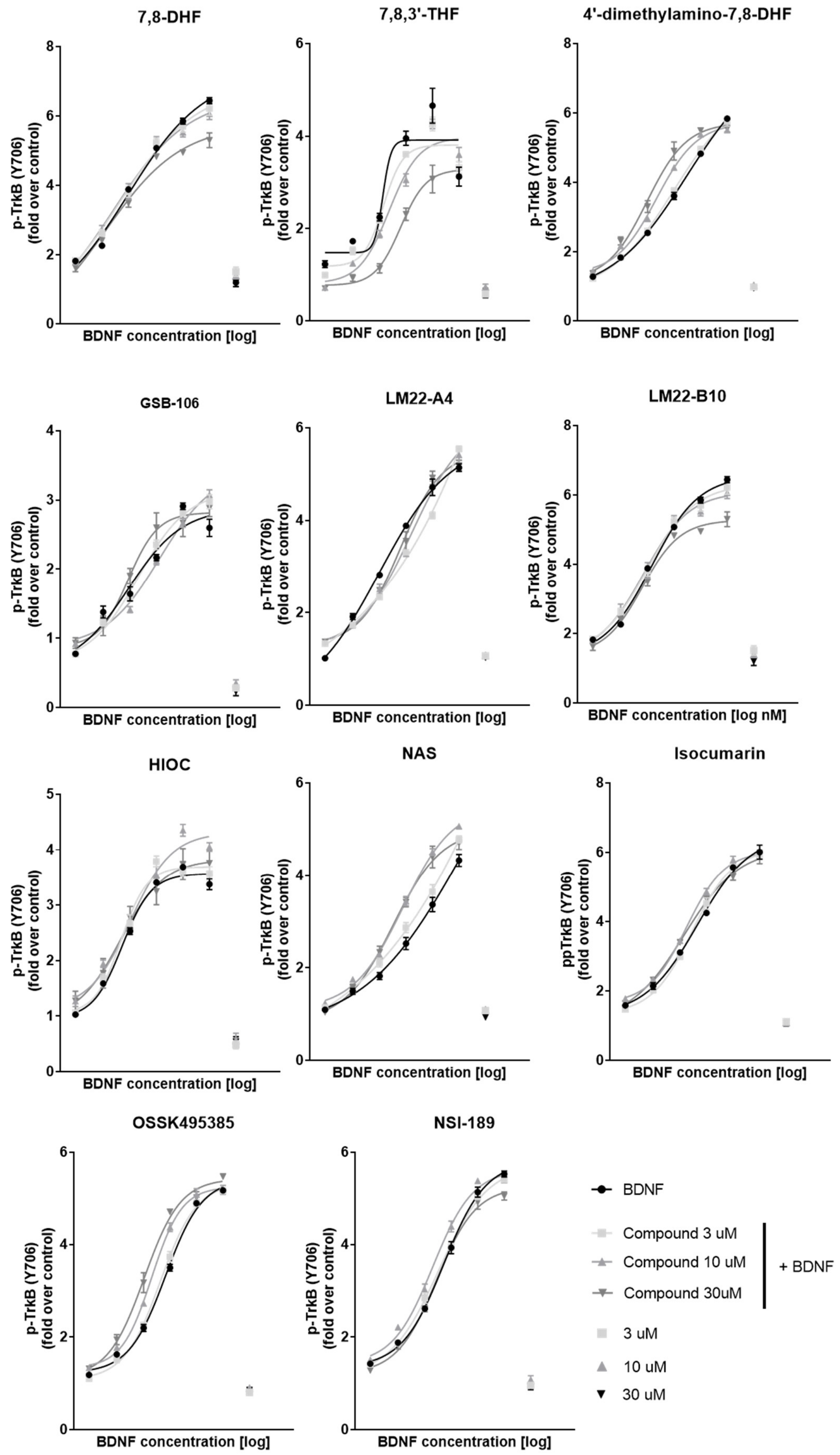

Figure S1. Results of compounds activity tested in PAM mode. Data are expressed as mean ± SEM, n=3.

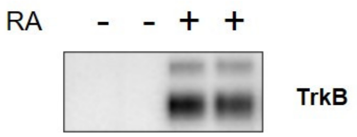

Figure S2. TrkB expression in RA-differentiated SH-SY5Y cell line.

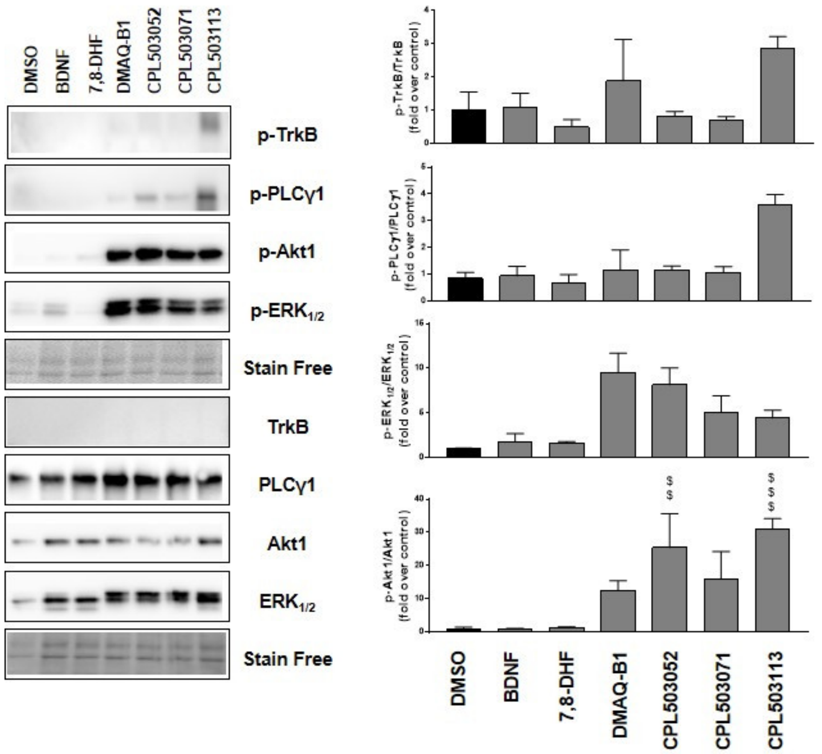

Figure S3. Effect of compound treatment in naïve SH-SY5Y cell line. Densitometry analysis of immunoblots. Bars represent mean ± SEM, n=3. Data was analyzed with one-way ANOVA followed by Dunnett post hoc test. $$ p < 0.01, $$$ p < 0.001 compared to DMSO treated group.

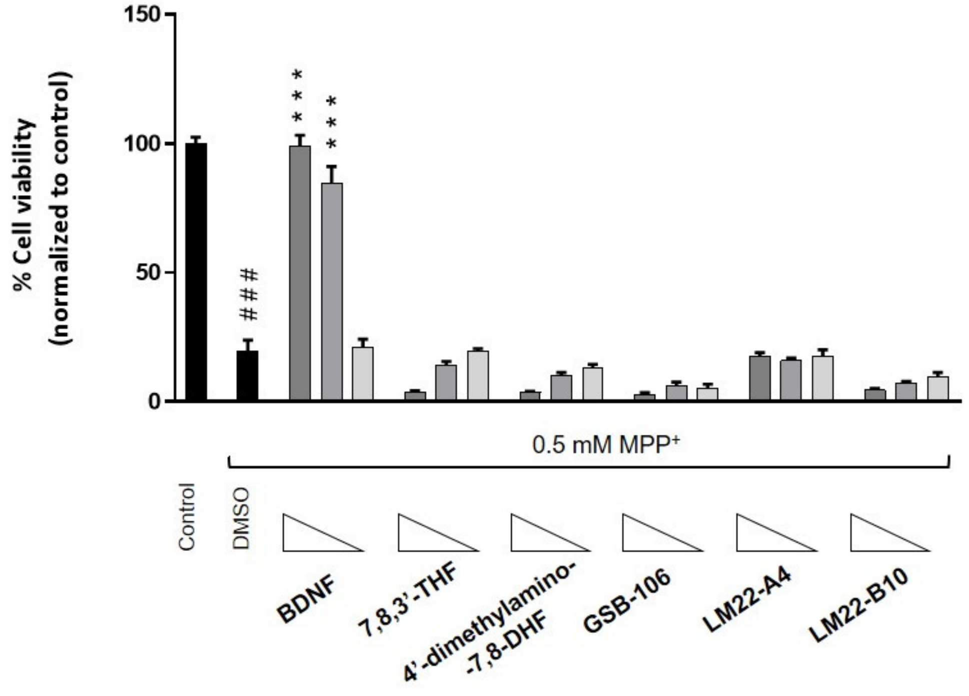

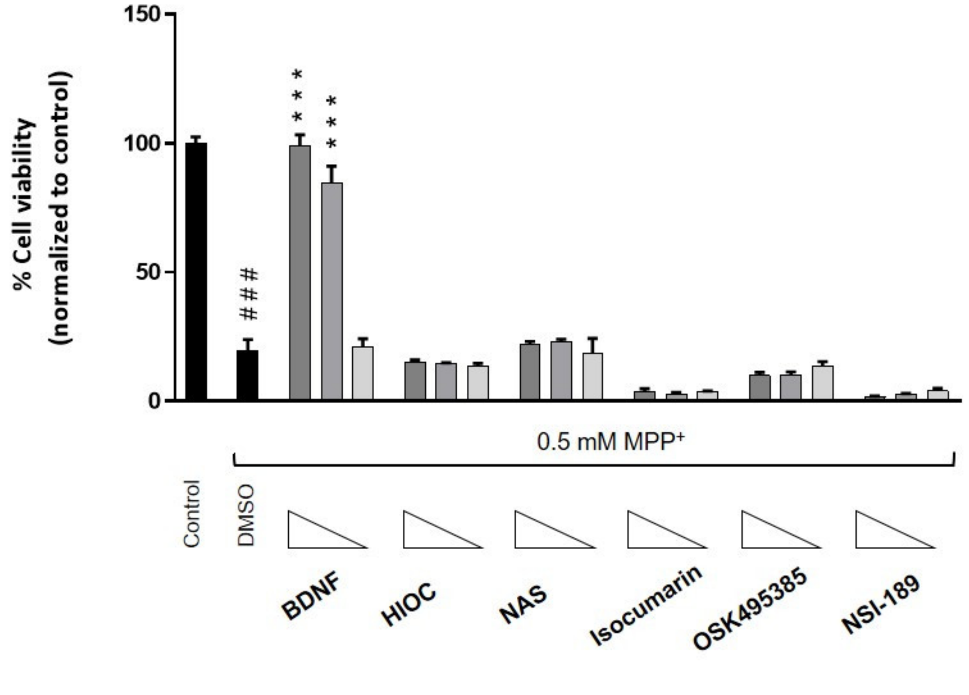

Figure S4 Effect of reference compounds on cytoprotection. Bars represent mean ± SEM, n=3. Data was analyzed with one-way ANOVA followed by Dunnett post hoc test. *** $p < 0.001$ compared to DMSO treated group. ### $p < 0.001$ compared to control group.

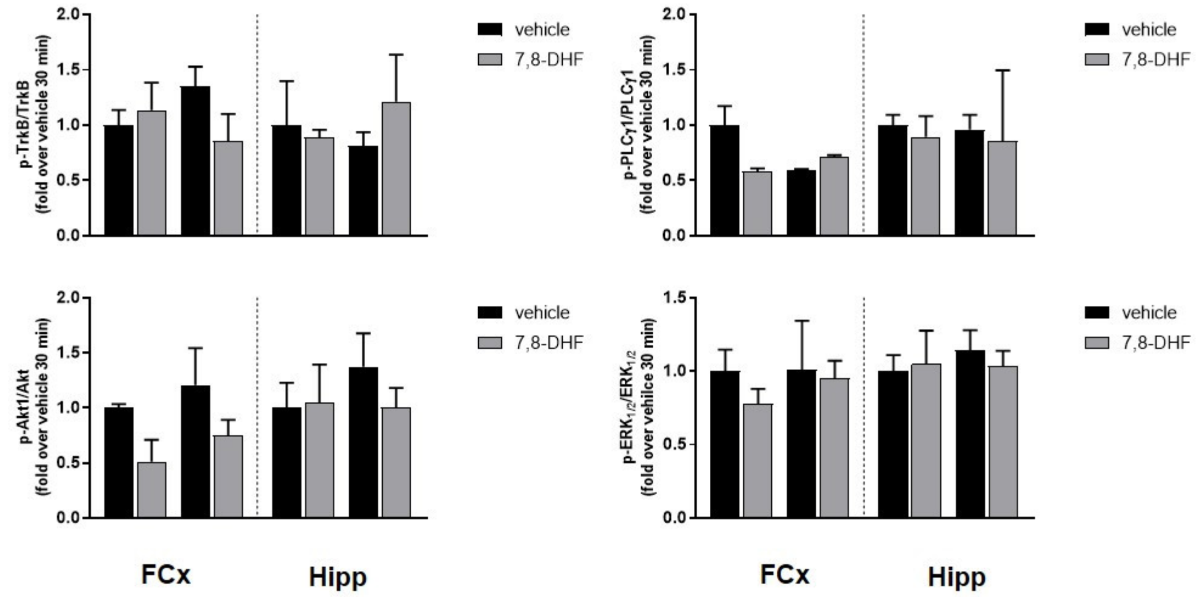

Figure S5 Densitometry analysis of protein phosphorylation in PD analysis. Bars represent mean ± SEM, n=4.

Table S2 Detailed results of analytes concentration in PK analysis. Metabolites (7-hydroxy-8methoxyflavone and 8-hydroxy-7-methoxyflavone).

		**Time**	**7,8-DHF** C [ng/mL] plasma C [ng/g] brain				**Metabolites** C [ng/mL] plasma C [ng/g] brain			
**7,8-DHF p.o 50mg/kg**	PLASMA	10 min	0,41	143,88	94,30	51,10	15,26	55,15	47,14	21,18
		30 min	15,94	21,89	29,57	6,37	32,57	41,17	22,05	9,04
		1h	<LLQ	<LLQ	<LLQ	<LLQ	3,56	10,78	13,44	0,53
		2h	<LLQ	<LLQ	<LLQ	2,21	24,72	22,61	8,25	21,07
		4h	<LLQ	<LLQ	<LLQ	<LLQ	4,48	9,43	12,29	17,09
	BRAIN	10 min	26,43	26,28	23,33	25,59	<LLQ	<LLQ	<LLQ	<LLQ
		30 min	11,80	35,36	14,06	2,03	<LLQ	<LLQ	<LLQ	<LLQ
		1h	6,45	<LLQ	<LLQ	<LLQ	<LLQ	<LLQ	<LLQ	<LLQ
		2h	0,91	<LLQ	21,70	<LLQ	<LLQ	<LLQ	<LLQ	<LLQ
		4h	<LLQ	<LLQ	<LLQ	<LLQ	<LLQ	<LLQ	<LLQ	<LLQ
**7,8-DHF i.v. 1mg/kg**	PLASMA	5min	<LLQ	<LLQ	<LLQ	<LLQ	21,37	13,14	20,04	12,34
		10 min	<LLQ	<LLQ	<LLQ	<LLQ	5,02	1,01	<LLQ	1,46
		30 min	<LLQ	<LLQ	<LLQ	<LLQ	4,05	0,92	0,59	0,00
		1h	<LLQ	<LLQ	<LLQ	<LLQ	3,13	<LLQ	<LLQ	<LLQ
		2h	<LLQ	<LLQ	<LLQ	<LLQ	<LLQ	<LLQ	<LLQ	<LLQ
	BRAIN	5min	<LLQ	<LLQ	<LLQ	<LLQ	<LLQ	<LLQ	<LLQ	<LLQ
		10 min	<LLQ	<LLQ	<LLQ	<LLQ	<LLQ	<LLQ	<LLQ	<LLQ
		30 min	<LLQ	<LLQ	<LLQ	<LLQ	<LLQ	<LLQ	<LLQ	<LLQ
		1h	<LLQ	<LLQ	<LLQ	<LLQ	<LLQ	<LLQ	<LLQ	<LLQ
		2h	<LLQ	<LLQ	<LLQ	<LLQ	<LLQ	<LLQ	<LLQ	<LLQ

<LLQ- below Lower Limit of Quantification

Table S3. BioPrint® Profile Panel results for 7,8-DHF

Target	Assay mode	Family	% of specific inhibition	Target	Assay mode	Family	% of specific inhibition
A1	Agonist	Receptor	99	CB1	Agonist	Receptor	1
xanthine oxidase/ superoxide O2- scavenging		Non-Kinase Enzyme	92	caspase-3		Non-Kinase Enzyme	1
MT3 (ML2)	Agonist	Receptor	86	HIV-1 protease		Non-Kinase Enzyme	1
COX2		Non-Kinase Enzyme	82	AR	Agonist	Receptor	0
A2B	Antagonist	Receptor	78	CDK2		Kinase	0
A2A	Agonist	Receptor	75	GR	Agonist	Receptor	-1
MMP-9		Non-Kinase Enzyme	72	glycine	Antagonist	Ion Channel	-1
BZD	Agonist	Ion Channel	71	norepinephrine transporter	Antagonist	Transporter	-1
A3	Agonist	Receptor	68	α2B	Antagonist	Receptor	-2
MMP-2		Non-Kinase Enzyme	56	motilin	Agonist	Receptor	-2
Lyn A kinase		Kinase	53	M2	Antagonist	Receptor	-2
5-HT2B	Agonist	Receptor	50	NK2	Agonist	Receptor	-2
LXRβ	Agonist	Receptor	49	μ (MOP)	Agonist	Receptor	-2
COX1		Non-Kinase Enzyme	49	V2	Agonist	Receptor	-2
PDE5		Non-Kinase Enzyme	48	5-HT3	Antagonist	Ion Channel	-2
PDE4D2		Non-Kinase Enzyme	38	GABA transporter	Antagonist	Transporter	-2
MAO-A	Antagonist	Non-Kinase Enzyme	35	ATPase (Na+/K+)		Non-Kinase Enzyme	-2
PDE6		Non-Kinase Enzyme	30	α1B	Antagonist	Receptor	-3
kainate	Agonist	Ion Channel	26	CB2	Agonist	Receptor	-3
Abl kinase		Kinase	25	5-HT6	Agonist	Receptor	-3
AT1	Antagonist	Receptor	24	PCP	Antagonist	Ion Channel	-3
CCK1	Agonist	Receptor	21	D1	Antagonist	Receptor	-4
PPARγ	Agonist	Receptor	21	H4	Agonist	Receptor	-4
Fyn kinase		Kinase	21	M1	Antagonist	Receptor	-4

MT1 (ML1A)	Agonist	Receptor	20	5-HT2A	Agonist	Receptor	-4
IP (PGI2)	Agonist	Receptor	17	5-HT2C	Agonist	Receptor	-4
FLT-1 kinase		Kinase	17	Ca2+ channel	Antagonist	Ion Channel	-4
CCK2 (CCKB)	Agonist	Receptor	15	5-HT transporter	Antagonist	Transporter	-4
BACE-1		Non-Kinase Enzyme	15	α2A	Antagonist	Receptor	-5
GABAA1 (h) (α1, β2, γ2)	Agonist	Receptor	12	β1	Agonist	Receptor	-5
IRK		Kinase	12	β2	Antagonist	Receptor	-5
BB3	Agonist	Receptor	11	GABAB(1b)	Antagonist	Receptor	-5
NK1	Agonist	Receptor	11	H1	Antagonist	Receptor	-5
NOP (ORL1)	Agonist	Receptor	11	MC1	Agonist	Receptor	-5
NMDA	Antagonist	Ion Channel	11	MC3	Agonist	Receptor	-5
PDE3B		Non-Kinase Enzyme	11	MC4	Agonist	Receptor	-5
MMP-1		Non-Kinase Enzyme	10	M4	Antagonist	Receptor	-5
Y1	Agonist	Receptor	8	δ(DOP)	Agonist	Receptor	-5
Estrogen Erα	Agonist	Receptor	7	B2	Agonist	Receptor	-6
AMPA	Agonist	Ion Channel	7	H3	Agonist	Receptor	-6
AT2	Agonist	Receptor	6	Ca2+ channel	Antagonist	Ion Channel	-6
FP	Agonist	Receptor	6	M3	Antagonist	Receptor	-7
CRF1	Agonist	Receptor	5	Cl- channel	Antagonist	Ion Channel	-7
N neuronal α4β2	Agonist	Receptor	5	5-HT1D	Agonist	Receptor	-8
choline transporter	Antagonist	Transporte r	5	ACE-2		Non-Kinase Enzyme	-8
PDE2A1		Non-Kinase Enzyme	5	UT	Agonist	Receptor	-9
ACE		Non-Kinase Enzyme	5	ETA	Agonist	Receptor	-10
acetylcholi nesterase		Non-Kinase Enzyme	5	CysLT1 (LTD4)	Agonist	Receptor	-10
APJ (apelin)	Agonist	Receptor	4	5-HT4e	Antagonist	Receptor	-11
CCR2	Agonist	Receptor	4	V1a	Agonist	Receptor	-11
5-HT7	Agonist	Receptor	4	Na+ channel	Antagonist	Ion Channel	-11

SKCa channel	Antagonist	Ion Channel	4	MCH1	Agonist	Receptor	-12
dopamine transporter	Antagonist	Transporter	4	VPAC1	Agonist	Receptor	-13
ZAP70 kinase		Kinase	4	D2S	Agonist	Receptor	-15
glucagon	Agonist	Receptor	3	Ca2+ channel	Antagonist (diltiazem site)	Ion Channel	-15
kappa	Agonist	Receptor	3	RARα	Agonist	Receptor	-16
5-HT1B	Antagonist	Receptor	3	N muscle-type	Antagonist	Receptor	-20
sigma	Agonist	Receptor	3	PAF	Agonist	Receptor	-20
inducible NOS		Non-Kinase Enzyme	3	CaMK2α		Kinase	-20
α1A	Antagonist	Receptor	2	sst4	Agonist	Receptor	-23
D3	Antagonist	Receptor	2	ERK2		Kinase	-23
TNF-α	Agonist	Receptor	2	β3	Antagonist	Receptor	-25
EP2	Agonist	Receptor	2	ETB	Agonist	Receptor	-26
sst1	Agonist	Receptor	2	5-HT1A	Agonist	Receptor	-26
Ca2+ channel	Antagonist (verapamil site)	Ion Channel	2	H2	Antagonist	Receptor	-37
Guanylyl cyclase	Activator effect		2	p38α kinase		Kinase	-50
α2C	Antagonist	Receptor	1				

Table S4. Chemical structure of compounds.

Name	Reference	Structure
7,8-dihydroxyflavone (7,8-DHF)	[12]	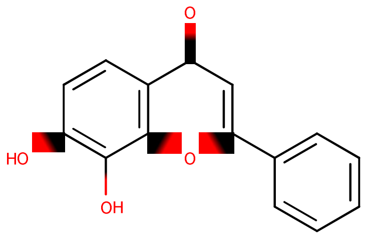
7,8,3′-trihydroxyflavone (7,8,3′-THF)	[13]	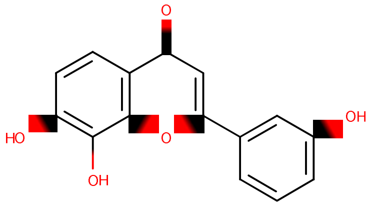
4′-dimethylamino-7,8-DHF (4′-DMA-7,8-DHF)	[13]	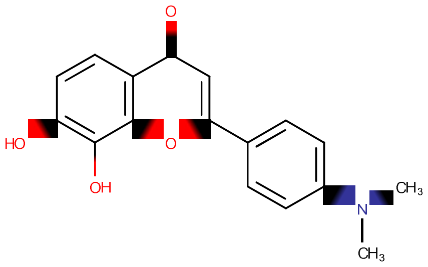
GSB-106	[19]	$[OH\text{-}Suc\text{-}Ser\text{-}Lys\text{-}NH\text{-}(CH_2)_3\text{-}]_2$
LM22-A4	[18]	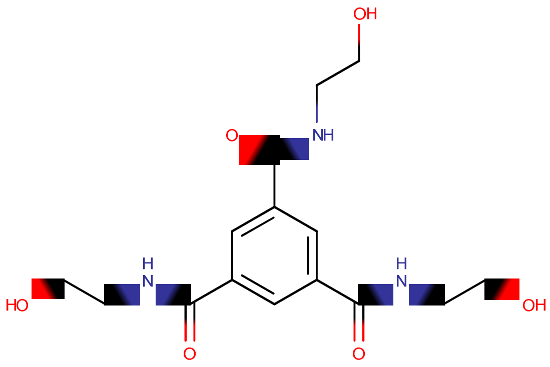
LM22-B10	[18]	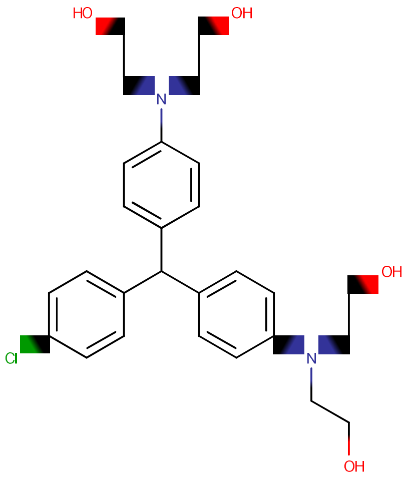

HIOC	[23]	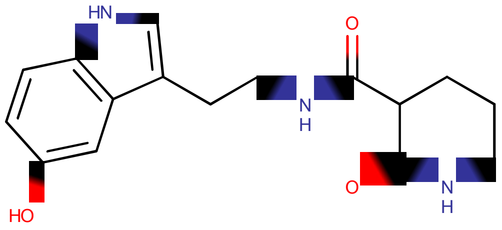
NAS	[20]	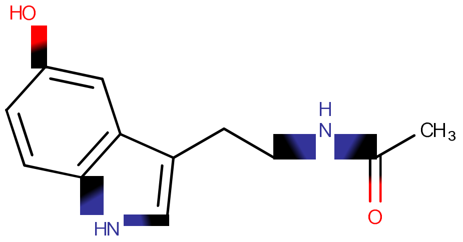
8-hydroxy-3-(4′-methoxyphenyl)-isocumarin (Isocumarin)	[21]	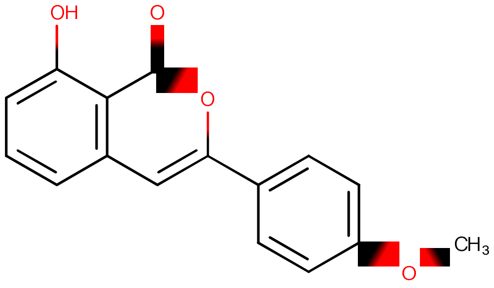
NSI-189	[22]	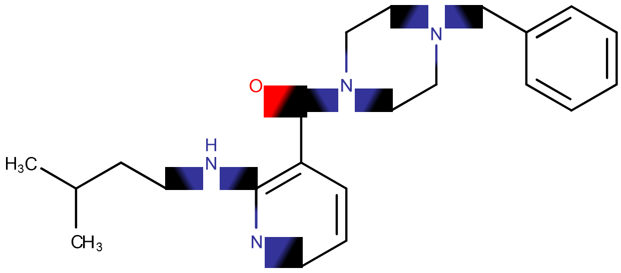
DMAQ-B1	[17]	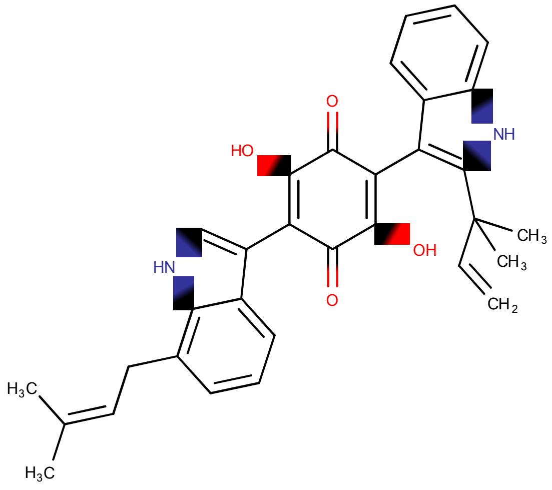

OSSK-495385	[24]	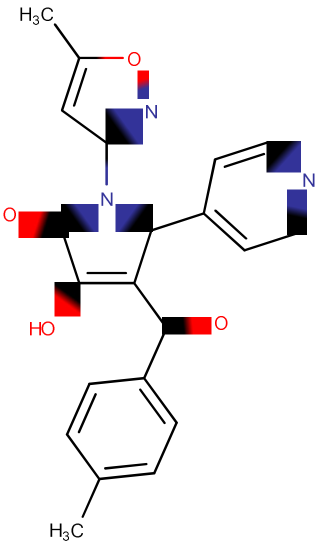
CPL503052 (didemethylasterriquinone D)	[46]	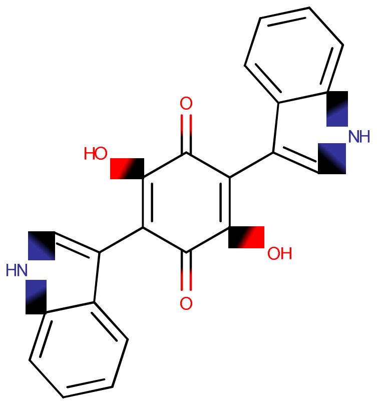
CPL503071	[46]	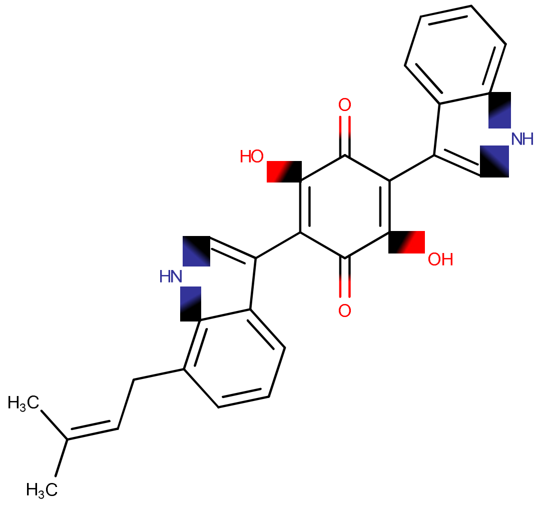

CPL503113	[47]	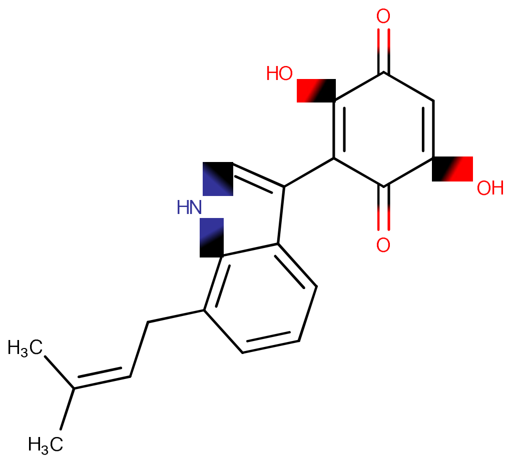
7-hydroxy-8-methoxyflavone (7H8M-flavone)	[29]	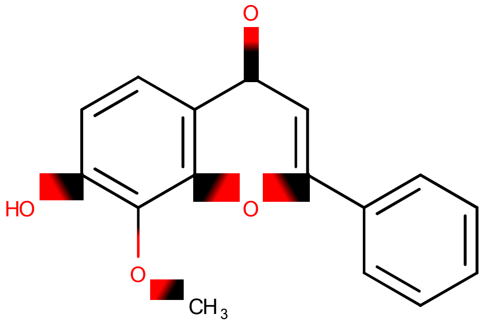
7-methoxy-8-hydroxyflavone (7M8H-flavone)	[29]	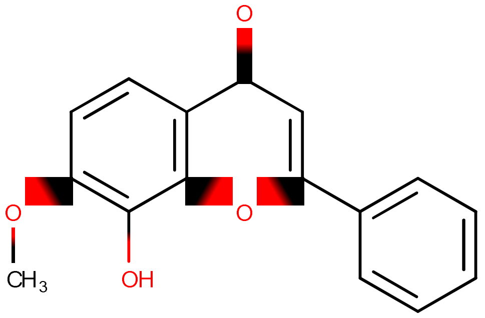
